# Disproportionate Cochlear Length in Genus *Homo* Shows a High Phylogenetic Signal during Apes’ Hearing Evolution

**DOI:** 10.1371/journal.pone.0127780

**Published:** 2015-06-17

**Authors:** J. Braga, J-M. Loubes, D. Descouens, J. Dumoncel, J. F. Thackeray, J-L. Kahn, F. de Beer, A. Riberon, K. Hoffman, P. Balaresque, E. Gilissen

**Affiliations:** 1 Hominid Evolutionary Biology, AMIS-UMR 5288 CNRS, University of Toulouse (Paul Sabatier), Toulouse, France; 2 Evolutionary Studies Institute, University of the Witwatersrand, Johannesburg, South Africa; 3 Statistics and Probabilities Team, Institute of Mathematics of Toulouse, UMR 5219 CNRS-Université de Toulouse (Paul Sabatier), Toulouse, France; 4 Institut d'Anatomie Normale et Pathologique, Faculté de Médecine de Strasbourg, Strasbourg, France; 5 South African Nuclear Energy Corporation, Pelindaba, North West Province, South Africa; 6 Laboratoire Evolution et Diversité Biologique, UMR 5174 CNRS, University of Toulouse (Paul Sabatier), Toulouse, France; 7 Royal Museum for Central Africa, Tervuren, Belgium and Laboratory of Histology and Neuropathology, Université libre de Bruxelles, Brussels, Belgium; Museo Nazionale Preistorico Etnografico 'L. Pigorini', ITALY

## Abstract

Changes in lifestyles and body weight affected mammal life-history evolution but little is known about how they shaped species’ sensory systems. Since auditory sensitivity impacts communication tasks and environmental acoustic awareness, it may have represented a deciding factor during mammal evolution, including apes. Here, we statistically measure the influence of phylogeny and allometry on the variation of five cochlear morphological features associated with hearing capacities across 22 living and 5 fossil catarrhine species. We find high phylogenetic signals for absolute and relative cochlear length only. Comparisons between fossil cochleae and reconstructed ape ancestral morphotypes show that *Australopithecus* absolute and relative cochlear lengths are explicable by phylogeny and concordant with the hypothetized ((*Pan*,*Homo*),*Gorilla*) and (*Pan*,*Homo*) most recent common ancestors. Conversely, deviations of the *Paranthropus* oval window area from these most recent common ancestors are not explicable by phylogeny and body weight alone, but suggest instead rapid evolutionary changes (directional selection) of its hearing organ. Premodern (*Homo erectus*) and modern human cochleae set apart from living non-human catarrhines and australopiths. They show cochlear relative lengths and oval window areas larger than expected for their body mass, two features corresponding to increased low-frequency sensitivity more recent than 2 million years ago. The uniqueness of the “hypertrophied” cochlea in the genus *Homo* (as opposed to the australopiths) and the significantly high phylogenetic signal of this organ among apes indicate its usefulness to identify homologies and monophyletic groups in the hominid fossil record.

## Introduction

Body size constraints, lifestyles, and other environment-related parameters moduling survival and reproduction, are key players in the evolution of mammalian features [1–4]. Because sensory organs influence optimal decisions when interacting with environmental signals, it is important to reliably assess their dependence on selection, scaling rules and phylogeny during evolution [5–7]. Since predictions of hearing capabilities in mammal species have been proposed from observations on gross features of the cochlea [8]—the auditory organ which plays the most important role in determining the bandwidth of hearing [9]—attention needs to be given on the relative roles of selection, interspecies allometry and phylogenetic relationships in shaping its evolution. In particular, it is important to use descriptive statistics to measure the tendency for evolutionarily related species to resemble each other in cochlear morphology due to their recent shared ancestry (i.e., the phylogenetic signal, as defined in [10]).

The presence of a phylogenetic signal implies that the topology and branch lengths of a given phylogenetic gene-based tree are proportional to the observed variance of evolution for a trait measured among a set of terminal species. Importantly, if the phylogenetic signal of a given trait is low or absent (i.e., phylogenetically-related species are not more similar than expected by chance), our ability to infer ancestral states of such an evolutionary malleable feature will be more limited. Therefore, measures of the phylogenetic signal represent a prerequisite for the study of evolutionary processes. This statistic allows comparisons of features in order to assess potential differences in patterns of evolutionary processes between them.

The phylogenetic signal has not been used to investigate evolutionarily the as yet unknown cochlear differences across the five lineages of living apes (*Homo*, *Pan*, *Gorilla*, *Pongo*, *Hylobates*) and of their fossil relatives, with their large range of developmental and reproductive strategies. The length of the cochlea has never been measured in early hominin specimens (australopiths and early *Homo*), and was found to be shorter than in modern humans [11]. Moreover, it was suggested that cochlear length provided a good estimate of low-frequency hearing in non-human primates [12,13]. If cochlear length is taken as a proxy measure of a shorter basilar membrane length in early hominins (with its sensors tuned to high frequencies at its base and lower frequencies progressively towards the apex), it could be interpreted as consistent with a better low-frequency sensitivity in humans as compared to the australopiths, with similarities between species perhaps associated with responses to similar environmental conditions (homoplasy). Indeed, genes involving hearing show convergent signals due to adaptive selection among some echolocating mammals [14], parallel accelerated rates of evolution in gorilla and human lineages [15], and positive selection in humans but not in chimpanzees [16]. These findings demonstrate the occurrence of homoplasy during mammals’ and apes’ hearing evolution, an obstacle to accurate identifications of monophyletic groups among fossil species.

In cases where morphology-based or gene-based analyses yield conflicting phylogenetic results due to homoplasy, phylogenetically-based statistical methods (as defined in [10]) offer possibilities to identify real homologies through measures of the phylogenetic signal contained in morphology. Moreover, since the nomenclature of living and fossil species requires the incorporation of information about both adaptive strategies and monophyly [17], an approach combining measurements of cochlear gross features with phylogenetically-based statistical methods [18,19] can help understanding key stages in hearing evolution, including in the most recent common ancestors (MRCAs) of apes, great apes (hominids) and hominins (who likely lived respectively in the periods between 23 to 16, 16 to 15 and 8 to 4 millions years ago—Myrs—as indicated through a combination of molecular and paleontological estimates [20, 21]), and at the origin of our human genus.

### The phylogenetic signal and monophyletic groups

Ape lineages have accumulated changes after their separation [15]. However, in the absence of proper distinctions of similarities due to shared recent history from homoplasies, it remains challenging to locate ape fossils on their correct monophyletic group [20,22]. Due to the occurrence of homoplasic features in the apes’ fossil record, it remains challenging to define more precisely several genera, including our own genus *Homo* [23,24]. For instance, comparative studies among several groups of mammals, including apes (hominoids), suggested that the masticatory system might represent a “homoplasy ghetto” [23–25]. Since cladistic analyses often provide the most parcimonious trees, they do not help identifying homoplasic features. Monophyly represents one of the two necessary conditions (in association with adaptative strategies) to identify accurately a genus or any other level of the biological nomenclature [17,23,24]. A descriptive statistic that assesses the strength of the phylogenetic signal of sets of traits (already widely used in ecological and evolutionary research) can be complimentary to cladistic investigations of apes’ evolutionary scenarios and to predictions of ancestral states. It can also improve classifications of fossil and living ape species into true monophyletic groups.

### Correlated trait evolution and interspecies allometry

It is also important to reliably estimate correlated evolution of characters while simultaneously estimating the strength of phylogenetic signal. For instance, the use of phylogenetically informed statistical procedures enhances the ability to detect species that deviate significantly from general allometric equations [26]. Predictions of ape’s hearing evolution made from cochlear morphological features have not been made to determine whether changes occurred primarily due to body mass increase through genetic/mechanistic interactions or were selected independent of body mass through ecological or functional processes yet to be identified. In the context of the evolution of hearing among apes, changes in body mass may entail changes in cochlear features that facilitated new behaviors. Indeed, the highest audible frequency for a mammal species is negatively correlated with body and ossicle mass, head size [27] and with the distance between the ears [28]. In ground-dwelling mammals, the frequency range of hearing is reflected by in the length of the cochear duct [5] which is in turn scaled with body mass [29]. However, the tonotopic organization of the cochlea [30] is complex and represents an intertwining of functional performances, developmental and genetic mechanisms [8,14,29,31] less prone to environmental pressures than the external/middle ears [31].

### Aims of the present study

**Fig 1:**
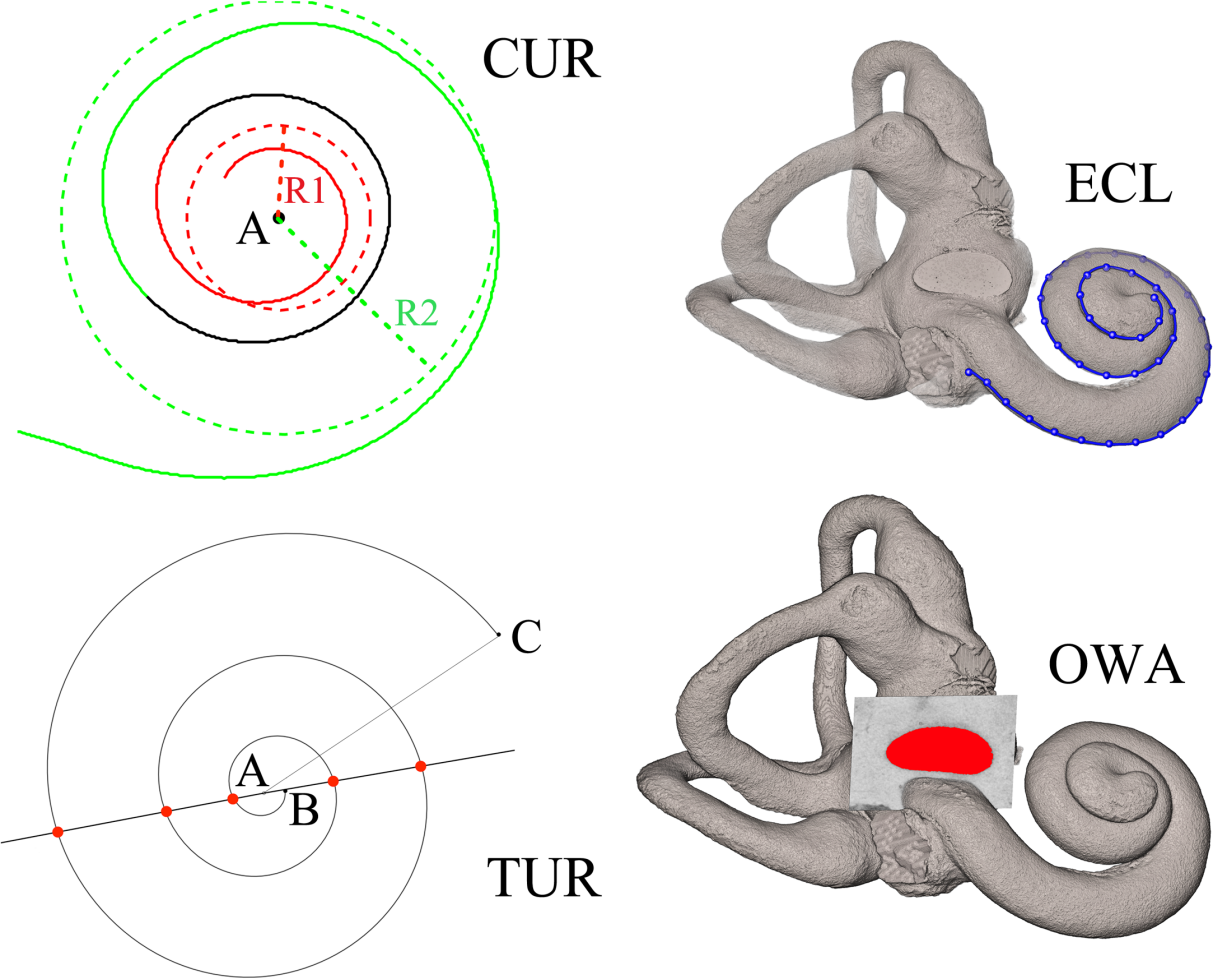
Illustrations of the five cochlear features investigated in this study and expressed as continuous variables. The external cochlear length (ECL, in mm), number of turns (TUR, expressed as the sum of full circle rotations and the angle between lines “AB”—center to apex—and “AC”—center to base), and relative length (RECL = ECL/TUR, in mm), the curvature gradient (CUR, expressed as a dimensionless ratio between the radii of the larger first—noted “R2”—and the smaller last spiral turns—noted “R1”), and the oval window area (OWA in mm^2^).

Here we used microfocus x-ray computed tomography (micro-ct) to investigate five vestibular/cochlear features (for simplicity, called “cochlear” throughout this paper) associated with hearing capacities [8,13,27,29,32]: the external cochlear length (ECL), number of turns (TUR), and relative length (RECL = ECL/TUR), the curvature gradient (CUR), and the oval window area (OWA) located near the cochlear entrance where the opening of the vestibular system attaches to the stapedial footplate (Fig 1). Our study sample was composed of all the five main apes’ living lineages (*Homo*, *Pan*, *Gorilla*, *Pongo*, *Nomascus*/ *Hylobates*). It also included fossil species (*Oreopithecus*, *Australopithecus*, *Paranthropus*, *Homo erectus* and Neanderthals) (Fig 1; for detailed information see S1 Table). We also investigate cochlear features in 13 living cercopithecoid species considered as outgroups. Our measurements were supplemented with literature data for non-catarrhine mammals and for three Middle Pleistocene humans from Spain often considered as early Neanderthals (OWA in fossils from Sima de los Huesos; S1 Table).

We first used an exploratory factor analysis to uncover the underlying relationships among the five cochlear variables and to identify the features that best explained the observed variability in our sample. We subsequently used these features and phylogenetic generalized linear models [10,18,19] to measure the strength of their phylogenetic signal and to determine whether some living species evinced cochlear shifts for their body mass after correcting for gene-based phylogeny. We also used Bayesian Markov chain Monte Carlo to reconstruct ancestral ape auditory conditions at each internal node of the hominoid phylogeny. We finally tested whether ape evolution shown in the fossil *Oreopithecus*, australopiths (*Australopithecus* and *Paranthropus*), *Homo erectus* and Neanderthals occurred in the direction predicted by our explicit allometric and phylogenetic analyses.

## Material and Methods

### Ethics statement

Our human and non-human samples were composed exclusively of dry skulls donated and curated in Museums from which we obtained permissions to access the specimens that have already been used in several published studies [11,33]. Therefore, no data reported here involved experimentation on subjects but only processing of micro-ct scans. The human skeletal sample is curated in the Institut d’Anatomie Normale et Pathologique of the University of Strasbourg. It was constituted mainly by Professors HWG Waldeyer (1836–1921) and G Schwalbe (1844–1916) before 1918 [34]. The non-human skeletal collections were elaborated in the early twentieth century from mostly wild-shot animals donated to the following museums: Muséum d’Histoire Naturelle de Toulouse (France), Musée Royal de l’Afrique Centrale (Tervuren, Belgium), Musée Zoologique de Strasbourg (France), Senckenberg Forschungsinstitut und Naturmuseum (Frankfurt, Germany). In all cases, the parties involved in the dissection of human cadavers or in the hunting of the animals held the proper permits.

### Samples and data collection

The inner ear is not notably influenced by postnatal growth and development [35], so that both juveniles and adults could be sampled and compared directly. We used micro-ct data obtained from dry skulls, or isolated petrous parts (pars petrosa) of the temporal bone, representing 86 juvenile and adult specimens of unknown sex and age. Sex diagnosis can be relatively straightforward in museum specimens representing adult dominant males of the two most dimorphic ape genera (*Gorilla* and *Pongo*). However, when museum specimens of unknown sex represent juveniles or non-dominant males, they cannot be sexed reliably.

The specimens were distributed among the following 9 hominoid and 13 cercopithecoid contemporaneous species: *Homo sapiens* (n = 22), *Pan paniscus* (n = 7), *Pan troglodytes* (n = 9), *Gorilla gorilla* (n = 7), *Pongo pygmaeus* (n = 8), *Nomascus concolor* (n = 1), *Hylobates moloch* (n = 1), *Hylobates lar* (n = 1), *Hylobates agilis* (n = 2), *Papio hamadryas* (n = 2), *Papio cynocephalus* (n = 5), *Papio ursinus* (n = 1), *Papio anubis* (n = 2), *Mandrillus sphinx* (n = 1), *Macaca radiata* (n = 1), *Macaca sylvanus* (n = 2), *Cercopithecus mona* (n = 1), *Cercopithecus hamlyni* (n = 1), *Cercocebus torquatus* (n = 1), *Colobus angolensis* (n = 3), *Colobus guereza* (n = 4), *Piliocolobus badius* (n = 4) (for detailed information see S1 Table). New and published [11] morphometric data were also obtained from adult hominoid specimens sampling five fossil taxa: *Oreopithecus bambolii* (BAC 208), a Mediterranean species which survived in isolation until 7.0–6.5 Myrs; *Australopithecus africanus* (STS 5) / *Australopithecus sp*. (StW 329, StW 98 and StW 255) from the late Pliocene deposits of the Sterkfontein site (South Africa), *Paranthropus robustus* (TM 1517, SK 879, SKW 18) and *Homo erectus* (SK 847) from the early Pleistocene sites of Kromdraai B (TM 1517) and Swartkrans (SK 879, SKW 18 and SK 847) (South Africa), and Neanderthals (Kr 38.20 and Kr 39.23) from the late Pleistocene site of Krapina (Croatia).

All but three specimens in our sample (*Oreopithecus* numbered BAC 208; and two Neanderthal specimens numbered Kr 38.20 and Kr 39.23) were obtained using five micro-ct systems (see details in S1 Table): the XtremeCT (Scanco Medical; http://www.scanco.ch) at the Institut de Médecine et de Physiologie Spatiales, Toulouse, France (http://www.medes.fr/); the Optiv CT160 (http://www.hexagonmetrology.fr) at Sematec Metrology, Oyonnax, France; the BIR ACTIS 225/300 from the Max Planck Institute for Evolutionary Anthropology in Leipzig (Germany); the X-Tek (Metris) XT H225L industrial CT system at the South African Nuclear Energy Corporation, Pelindaba (NECSA, www.necsa.co.za); and the Nikon Metrology XTH 225/320 LC dual source industrial CT system at the Palaeosciences Centre in the University of the Witwatersrand, Johannesburg (www.wits.ac.za/microct). The micro-ct data set for the *Oreopithecus* specimen was made available at: http://www.geo.unifi.it/ricerca/bambolii.htm) [36]. The micro-ct data set for the two Neanderthal specimens was made available at www.nespos.org. To the exception of the STS 5 skull (scanned at an isometric voxel size of 76.15 microns, μm), all the micro-ct data had isometric voxel dimensions ranging from 7.0 to 41.0 μm (S1 Table), hence allowing a good visualization of the cochlear structures. These relatively small voxel dimensions could be obtained due to the use of small skulls (mainly juvenile specimens) and isolated petrosals.

### Measurement methodology

All measurements were calculated using Matlab R 2012a (7.14, Mathworks). We first imported the μCT into the Avizo software package (www.vsg3d.com/avizo) for the 3D reconstruction of the air filled cochlea and oval window fossa. The ECL was measured by placing landmarks at small intervals along the outer circumference of the cochlea, between the cupula (apex) and the point marking the origin of the basal turn, where there is a saddle between the cochlear part and the vestibule, very close to the inferior margin of the round window (Fig 1). This method has already been used [13] making our measurements directly comparable. The ECL was used as a proxy for the length of the basilar membrane (as suggested in [13]) even if this measurement method likely overestimated the true length of the basilar membrane.

The number of turns (TUR) and the curvature (CUR) were also expressed as continuous variables obtained (using Matlab) from the coordinates of the landmarks placed on the outer circumference of the cochlea (Fig 1). We first computed the center of the cochlear spiral (noted “A”, Fig 1) from the local chords defined by the landmarks placed at its two extremities. This method has already been used [37]. We then calculated the equations of the two circles best fitted to the landmarks placed respectively on the first and last spiral turns and centered on “A” (shown in red and green, Fig 1). The CUR values corresponded to the ratio between the radii of the larger and the smaller circle (noted respectively “R2” and “R1”, Fig 1). We finally defined two distinct lines joining the center “A” with the two extremities of the spiral. The TUR values corresponded to the sum of full circle rotations and the angle between the two lines (Fig 1). As for ECL values, the TUR and CUR parameters were rounded to the nearest tenth.

The OWA was visualized in 3D after extracting an isosurface of its fossa (Fig 1). An oblique slice visually considered to best-fit the complete outline of the oval window was reconstructed. The OWA was then measured from its segmentation on this oblique slice. No data on ECL, RECL, TUR and CUR in non-human living hominoids have been published so far.

### Body mass

In order to investigate how interspecific differences in auditory structures may be caused by allometry, we compiled body mass data in living taxa [38–40] by averaging adult male and adult female values. Thus, we did not take into account interspecific differences in sexual dimorphism. Average weights may be overestimated in the particular case of genera in which sexual dimorphism is marked, like for instance *Gorilla*, *Pongo* and *Papio*. However, a twofold error in estimating body mass will cause a shift in log values of less that 10% of the entire range of data. Moreover, for most taxa, sufficient data on sexual dimorphism of the auditory structures are not yet available to permit a closer investigation of the interspecific allometric relationships. For fossil species we used estimates of body mass: 32 kg for *Oreopithecus* [41], 35.5 and 36 kg for respectively *Australopithecus* and *Paranthropus* [42], and 42 kg for early *Homo erectus* [42].

### Exploratory factor analysis

**Fig 2:**
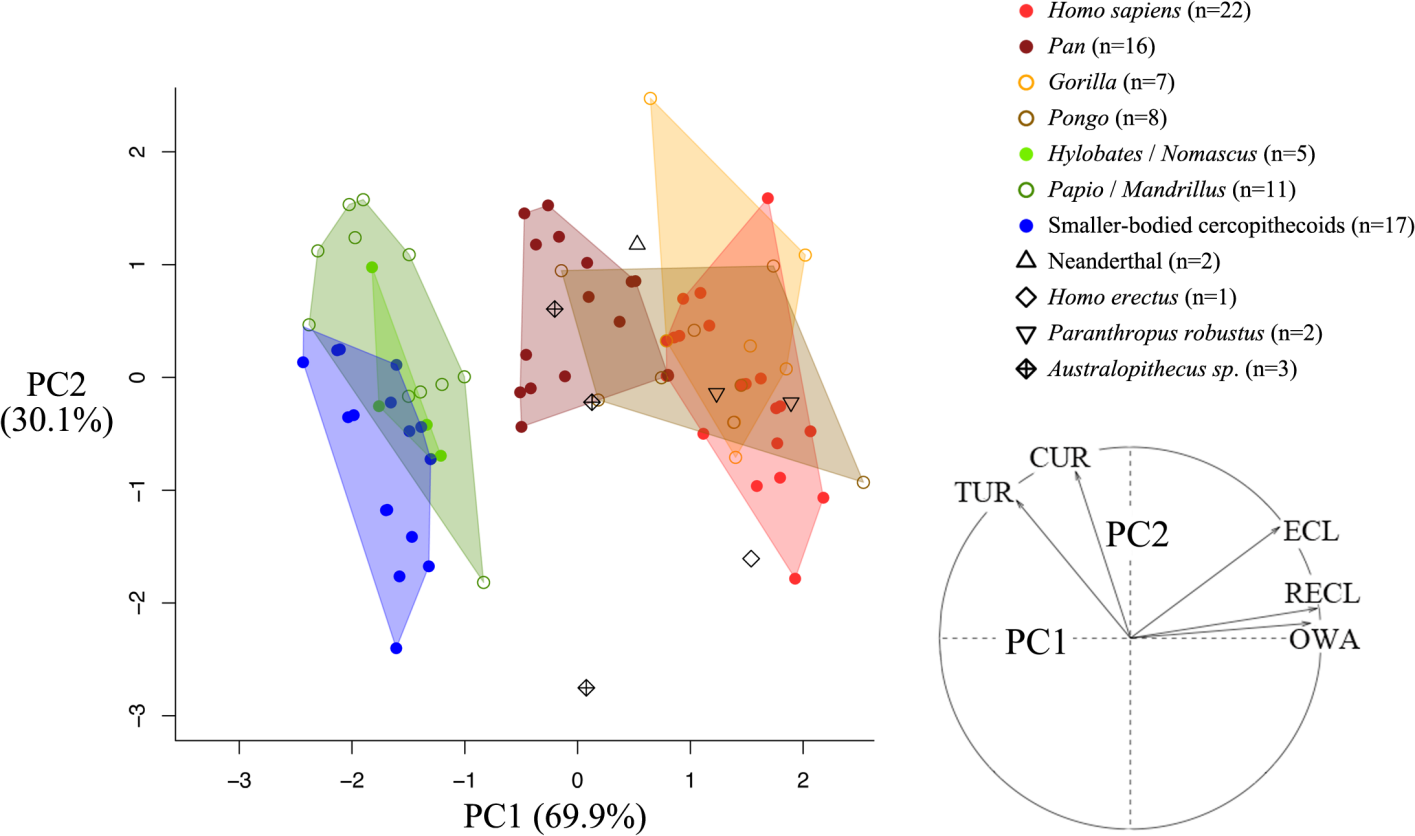
Factorial analysis of all five cochlear features illustrated in Fig 1 and principal component analysis of only RECL, CUR and OWA (to prevent redundancy) measured among hominoid living species and fossil taxa.

We performed principal component analyses (PCA) (Fig 2) in order to investigate the relationships and the hierarchy among our set of five continuous variables. A factor map allowed us to identify the cochlear features which played the most significant roles in cochlear variation among our sample of catarrhine species (Fig 2).

### Inter-species differences

We evaluated interspecies cochlear differences using a Monte Carlo permutation test (with the RStudio free software, Version 0.96.331) and the p-values for two samples t-tests applied to each pair of species (Table 1). For the randomization tests, we assumed that the cumulative distribution functions for the two samples were identical under the null hypothesis H0. The significance level was set at 5%.

**Table 1:** Interspecies RECL and OWA differences using a Monte Carlo permutation test with p-values for two samples t-tests applied to each pair of hominoid species.

		RECL comparisons								
		Hs	Pp	Pt	Gg	Pop	Hy	Nea.	Aus.	Par.
OWA comparisons	Hs		.01964*	.01894*	.27665	.05126	.03945*	.47569	.08479	.90663
	Pp	.01287*		.08173	.01784*	.05892	.04382*	.14436	.35448	.90793
	Pt	.0264*	.00765*		.04879*	.30664	.03908*	.19805	.3218	.16644
	Gg	.16406	.00518*	.01836*		.09212	.03044*	.36194	.08297	.48213
	Pop	.3061	.00519*	.04281*	40426		.03616*	.24662	.23918	.23438
	Hy	.04*	.0384*	.03741*	.03439*	.04414*		.11314	.03598*	.05942
	Nea.	-	-	-	-	-	-		.08408	.31871
	Aus.	.00321*	.37344	.41298	.02141*	.02323*	.02031*	-		.12177
	Par.	.07749	.00000*	.00000*	.51932	.46965	.00000*	-	0.15348	

Hs, *Homo sapiens*; Pp, *Pan paniscus*; Pt, *Pan troglodytes*; Gg, *Gorilla gorilla*; Pop, *Pongo pygmaeus*; No, *Nomascus sp*.; Hy, *Hylobates sp*.; Nea., Neanderthals; Aus., *Australopithecus sp*.; Par., *Paranthropus robustus*.

* indicates significant differences (at 5%).

### Catarrhine consensus phylogenetic tree

We used 10kTrees for Primates, V2 (http://10ktrees.fas.harvard.edu/) [43] to download the 50% majority rule catarrhine consensus tree (i.e., with nodes present on 50% or more of all trees) with ‘molecular-calibrated’ branch lengths (phylogram) rather than a time-calibrated ultrametric tree (chronogram, i.e. with path lengths identical for all species). We made this choice because of evidence for changes in mutation rate in primate evolution on large timescales, including an approximately 30% branch length decrease in humans compared to baboons since their common ancestor [44].

### Phylogenetic signal: Brownian model of evolution and Pagels’ λ

The phylogenetic signal measures the statistical dependence among observations for species related by a phylogenetic tree. The basic principle is to test whether a given tree better fits a set of species data observed at its tips as compared with the fit obtained when the same data have been randomly permuted across the tips (i.e., when the topology of the tree is destroyed). Our test was implemented via a phylogenetic generalised least squares (PGLS) approach. In PGLS mode, the phylogenetic tree is converted into a variance-covariance matrix, with the diagonal elements reporting the path length for each species (the root-to-tips distances; the variance) and the off-diagonal elements reporting the time of shared evolution for each pair of species (the distances from the root to the most recent common ancestor of each pair of species; the covariance). The covariance between the values in two tips of the tree is defined as the product of the trait values for the two tips, each measured as deviations from the ancestral state at the root node of the phylogeny. When two tips share a greater proportion of common history, their expected phylogenetic covariance is relatively high.

In order to compute the phylogenetic covariances, we used the most common model for the evolution of continuously valued traits: the Brownian model [10]. Under this model, the expected variance for the trait value at a given tip of the tree is directly proportional to the summed branch length from the root to that tip. Therefore, the expected covariance between two values at the tips of the tree is directly proportional to the shared history of the taxa represented by the two tips.

We used the likelihood ratio test to determine whether a Brownian model fitted our data and to compare two models of evolution: (i) a model that correspond to a standard Brownian constant-variance random-walk model with one parameter (variance of evolution) and (ii) a directional random-walk model with two parameters (variance of evolution and a parameter that reflects the degree of directional change). The likelihood ratio test compares the log-likelihood of the null hypothesis model (no directional trend exists) to that of the alternative hypothesis model (a directional trend exists) (S2 Table).

To measure the phylogenetic signal, we used the parameter lambda (λ) (Pagels’ λ) (S3 Table) which is multiplied to each off-diagonal elements in the variance-covariance matrix of shared evolutionary time between any pair of species in the gene-based phylogeny. The λ parameter reveals whether the phylogeny correctly predicts the patterns of covariance among species on a given trait, and its value can differ for different traits on the same phylogeny. The Pagels’ λ statistics varies between λ = 0 (the tree becomes more "star" like with all the branches emanating from a common node) and λ = 1 (the original tree is recovered). The λ parameter is typically estimated to obtain a value that maximizes the likelihood of the data. The statistical tests for a phylogenetic signal are successively performed under the null hypotheses that λ = 0, and that λ = 1. We reported tests for significant departure of λ from 0 and 1 (S3 Table). The procedure is as follows: (i) we estimate the maximum likelihood (ML) value of λ in our data, and get the log- likelihood of this model; (ii) we run a model with λ fixed at its maximum value of 1; (iii) we use a likelihood ratio test to decide whether a model with ML λ fits the data better than a model with λ = 1. This tells us whether the phylogenetic signal in the data is equal or less than expected under the Brownian model given the phylogeny; (iv) we repeat the procedure and compare the model with ML λ with one in which λ = 0. A likelihood ratio test of a model with ML λ versus a model with λ = 0 will tell us if the phylogenetic signal in the data is greater than 0.

The likelihood ratio test is calculated as: 2 * (log-likelihood of best fitting model—log-likelihood of worst fitting model). The best fitting model has the highest likelihood. The likelihood ratio is the absolute (i.e. positive) value of the difference between Log-likelihoods of the two competing nested models. We assess the significance of this value against a χ^2^ distribution with degrees of freedom (df) equal the difference in the number of estimated parameters between competing models (the directional random-walk model has two parameters and the Brownian motion model has one parameter). If the result is significant, then the directional random-walk model describes the data significantly better than the Brownian model, and should therefore be preferred, for instance when estimating ancestral states.

More details can be found in Blomberg et al. [10], Revell et al. [45] and http://www.anthrotree.info/wiki/projects/pica/The_AnthroTree_Website.html [46]. All the tests were made for each trait (cochlear parameters and body mass) separately on: (i) the entire sample of 22 catarrhines available in this study, (ii) the hominoid clade only (9 species), (iii) the cercopithecoid clade only (13 species). Because these calculations can be difficult when made using small numbers of species (i.e., less than 20), we consider our results based on all catarrhine species as more robust. We used the Unix executable program BayesTraits, Version 1 [47] (www.evolution.rdg.ac.uk/BayesTraits.html).

### Non-phylogenetic and phylogenetic interspecific linear regressions

We used both traditional (non-phylogenetic) and phylogenetic bivariate and multivariate linear regressions to investigate the relationship between log-transformed mean species values for cochlear parameters and body mass considered as the independent variable (for detailed information see S1 Text). We determined whether the variation in each cochlear parameter was conceived as being tied to, or best expressed as the variation in body mass. The non-phylogenetic regressions (S4 Table) and the Akaike information criterion (AIC) [48] were computed using the RStudio free software (Version 0.96.331R) while the phylogenetic regressions (S5 Table) were computed using Bayes Traits (Version 1).

In the non-phylogenetic approach, we used two distinct methods to get meaningful inter-species allometric information out of our data: the p-values of linear regressions and the Akaike information criterion (AIC) (S1 Text). The AIC method is a measure of the relative quality of a statistical model, for a given set of data, and provides a mean to select the best model. We also applied least-squares (LS) and reduced major axis (RMA) line-fitting techniques to our species mean data. The advantage of RMA regression is that unlike the LS one, it does not assume that the independent variable (x-variable) is measured without error [49]. Then, as body mass is taken as the independent variable, the RMA residuals for cochlear parameters will be biased in the same direction. Both LS and RMA regression equations have been calculated for the entire sample of catarrhines. Moreover, to test for potential grade shifts within catarrhines, we examined the scaling of cochlear parameters within cercopithecoids and hominoids separately. The significance level was set at 5%. Residual analyses were used to discriminate between species that had cochlear values deviating from the catarrhine inter-species allometric plan. We evaluated graphically how well the non-phylogenetic linear bivariate models fitted the data and how the data met the assumptions of the linear model. To evaluate deviations from the linear model assumptions we examined various diagnostic plots (S1 Text, S6 Table).

In the phylogenetic approach, we investigated whether accounting for phylogeny impacted on the estimates of the slope of cochlear traits on body weight, by using phylogenetic generalized least-squares (PGLS) bivariate and multiple regressions. The λ parameter was estimated while simultaneously calculating the correlation. We explored the effects of specific variables on the explanatory power of the models by statistically comparing models with versus without the variables in question using the log-likelihood ratio (LR) test (see above).

### Ancestral states reconstructions

We used Bayesian Markov chain Monte Carlo (with BayesTraits V1) to estimate cochlear trait changes at each internal nodes in the hominoid tree while accounting for the gene-based phylogeny (for detailed information see S1 Text). The three parameters of the phylogenetic tree were: τ, the topology of the tree; υ, the vector of branch lengths on the tree; σ the variance of the Brownian model of evolution. We made the bayesian phylogenetic inferences of three cochlear traits (ECL and RECL—with their high phylogenetic signal—and OWA), and body weight, from the posterior probability of the phylogenetic tree given the data matrix.

Estimates required two main steps. The first was to obtain a Markov chain Monte Carlo sampling of posterior probabilities of the Brownian model parameters: λ (phylogenetic signal) and α (phylogenetically weighted mean of the tip values falling within the species’s under a Brownian model) and the Brownian model constant variance. The second step was to use the Brownian motion model parameters to estimate posterior distribution values only for cochlear trait with a high phylogenetic signal (S7 Table). Deviations of fossil values from each ECL, RECL and OWA ancestral state distribution at all internal nodes on the hominoid tree were examined by Z-scores (S7 Table).

## Results

### Cochlear variation among living hominoid apes

We first perform a PC analysis with all the five cochlear variables. The first two principal components (PC1 and PC2) explain 62.6% and 28.6% of the variation respectively. The scores along PC1 provide a clear separation between living hominids one the one hand, and living hylobatids/cercopithecoids on the other (S1 Fig). Since RECL represents a combination of ECL and TUR, in order to prevent redundancy, we also produce a PCA using only RECL, OWA and TUR (Fig 2). In this case, we also observe a separation between hominids and hylobatids/cercopithecoids along PC1 (Fig 2). All fossil hominins group with living hominids along PC1 (Fig 2). The factorial analysis shows that ECL, RECL and OWA are positive correlated with PC1, whereas RECL and OWA have the highest correlation (Fig 2). TUR and CUR are positively correlated with PC2 (Fig 2). Therefore, longer RECL and larger OWA are taxonomically useful to distinguish living hominids and fossil hominins from the other catarrhine species showing shorter RECL and smaller OWA.

**Fig 3:**
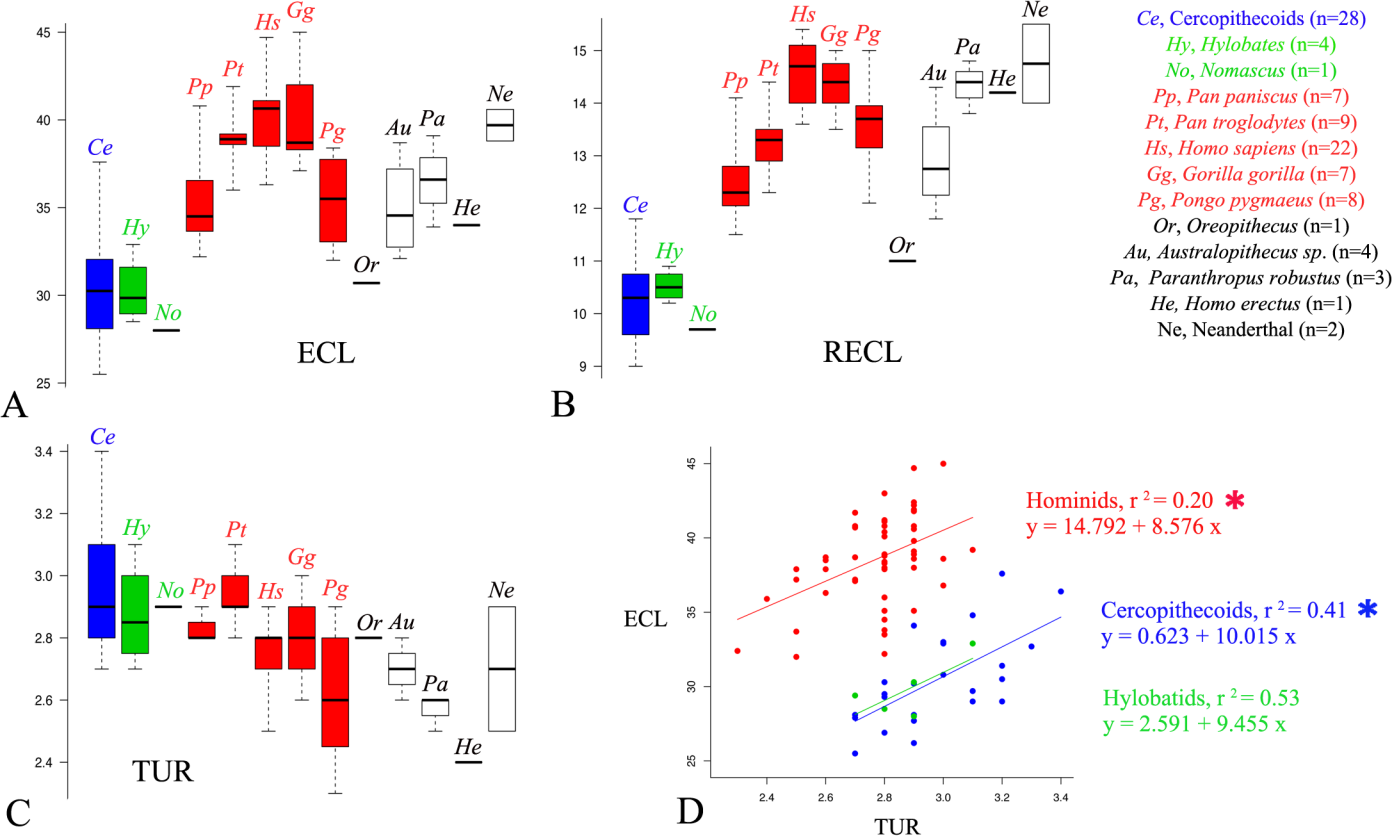
Micro-ct values of ECL (in mm) (A), RECL (in mm) (B) and TUR (C) among hominoid living species and fossil taxa. Summary statistics include sample size (n), the median (bold trait), the 0.25 and 0.75 quartiles, the maximum and minimum values. (D) Bivariate non phylogenetic interindividual linear regressions between ECL and TUR in hominids (filled red circles, n = 53), hylobatids (filled green circles, n = 5) and cercopithecoids (filled blue circles, n = 28) (S4 and S5 Tables). Significant correlation is indicated by * (P≤0.05).

**Fig 4:**
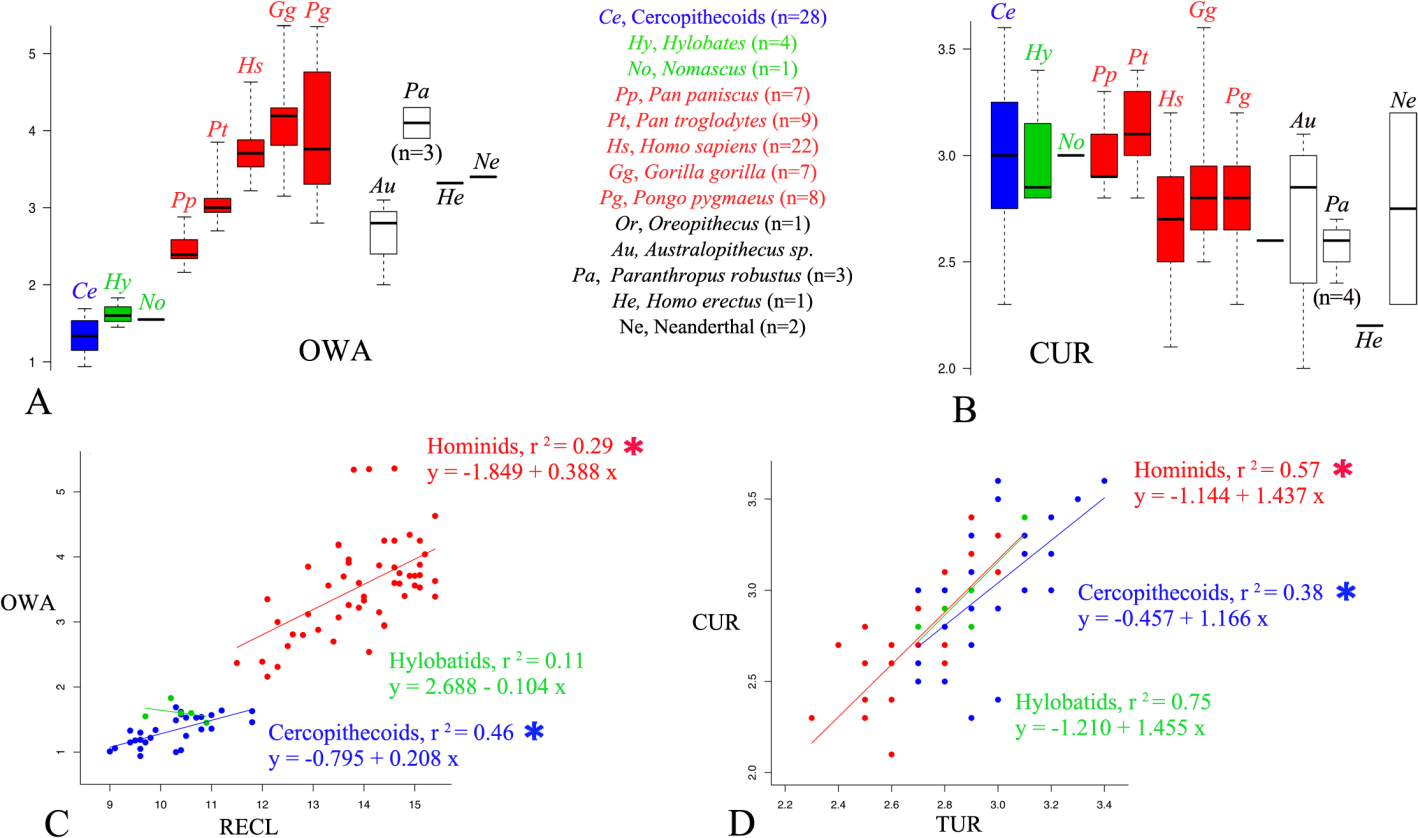
Micro-ct values of OWA (in mm^2^) (A) and CUR (B) among hominoid living species and fossil taxa. Summary statistics include sample size (n), the median (bold trait), the 0.25 and 0.75 quartiles, the maximum and minimum values. Bivariate non phylogenetic interindividual linear regressions between RECL and OWA (C), between TUR and CUR (D) in hominids (filled red circles, n = 53), hylobatids (filled green circles, n = 5) and cercopithecoids (filled blue circles, n = 28) (S4 and S5 Tables); Values for the OWA in Neanderthals were taken in Martinez *et al*. (2004) (S1 Table). Significant correlation is indicated by * (P≤0.05).

The variability sampled within and between living species for each cochlear trait confirms the better taxonomic usefulness of RECL and OWA as compared to ECL, TUR and CUR. All permutations tests reveal that shorter RECL (Fig 3) and smaller OWA (Fig 4) values measured in living *Pan* and *Hylobates* differ significantly from those observed in all other larger-bodied hominid species, except for the *Pan*-*Pongo* RECL comparison (Table 1). Only the larger RECL and OWA values observed in living hominids and in fossil hominins show almost no overlap with hylobatid and cercopithecoid data (Figs 3 and 4).

Non-phylogenetic controlled regressions confirm the unique cochlear morphological pattern among living hominids. The significant linear correlation between TUR and ECL indicates two distinct regression lines and shows that for any given TUR value, the hominid cochlea is longer than in hylobatids/cercopithecoids (Fig 3). The linear correlation between RECL and OWA is also significant, with two distinct regression lines separating hominids with their higher RECL and larger OWA one the one hand, from hylobatids and catarrhine monkeys on the other (Fig 4). The significant linear correlation between TUR and CUR (Fig 4) confirms the results already shown in the factor map (Fig 2).

Unfortunately, as yet patterns of ape sexual size dimorphism are difficult to investigate from museum collections because the available samples (particularily, juvenile specimens) cannot be fully exploited in the frequent absence of sex determinations [50]. Consequenty, for each nonhuman species, we calculate combined-sex coefficients of variations (CVs) only (S1 Table). The RECL CVs are nearly identical between hominoid species (ranging from 0.04 to 0.07; S1 Table). This is not the case for the OWA, with its smaller CVs in *Homo sapiens*, *Pan troglodytes* and *Pan paniscus* (0.09, 0.12 and 0.10, respectively) as compared to *Gorilla* and *Pongo* values (0.16 and 0.24, respectively). Interestingly, in these two latter highly sexually size dimorphic genera only, combined-sex OWA CVs increase four times when compared to RECL CVs. Permutations tests reveal non-significant differences between female and male *Homo sapiens* RECL (p = 0.349) and OWA (p = 0.247) (S1 Table). Moreover, modern human female and male CVs for both RECL and OWA are nearly similar to the combined-sex CVs, hence confirming no sexual dimorphism for these two cochlear traits (S1 Table).

### Phylogenetic signal and correlated trait evolution

When we perform the likelihood ratio tests to decide whether our data fit a Brownian constant-variance model of evolution or a directional random-walk model, we observe that all the p-values but one are not significant (S2 Table). Thus, the Brownian motion model should be preferred. This implies that the maximum likelihood estimate of the root node of the catarrhine tree can be reconstructed to lie within the range of values observed in our data.

Only for ECL and RECL we obtain a high phylogenetic signal for both hominoids and cercopithecoids considered separately. When we consider the sample of catarrhine species, we measure a close to one Pagel’s λ (respectively 0.91 and 0.93) significantly different from 0 (ECL: p = 0; RECL: p = 0), but not significantly different from the maximum value of 1 (RECL: p = 0.108; RECL: p = 0.073) (S3 Table). Importantly, contrary to the case when RECL is considered in the sample of catarrhines species, OWA shows a Pagel’s λ significantly different from 1 at the 5% level (p = 0.000) (S3 Table).

**Fig 5:**
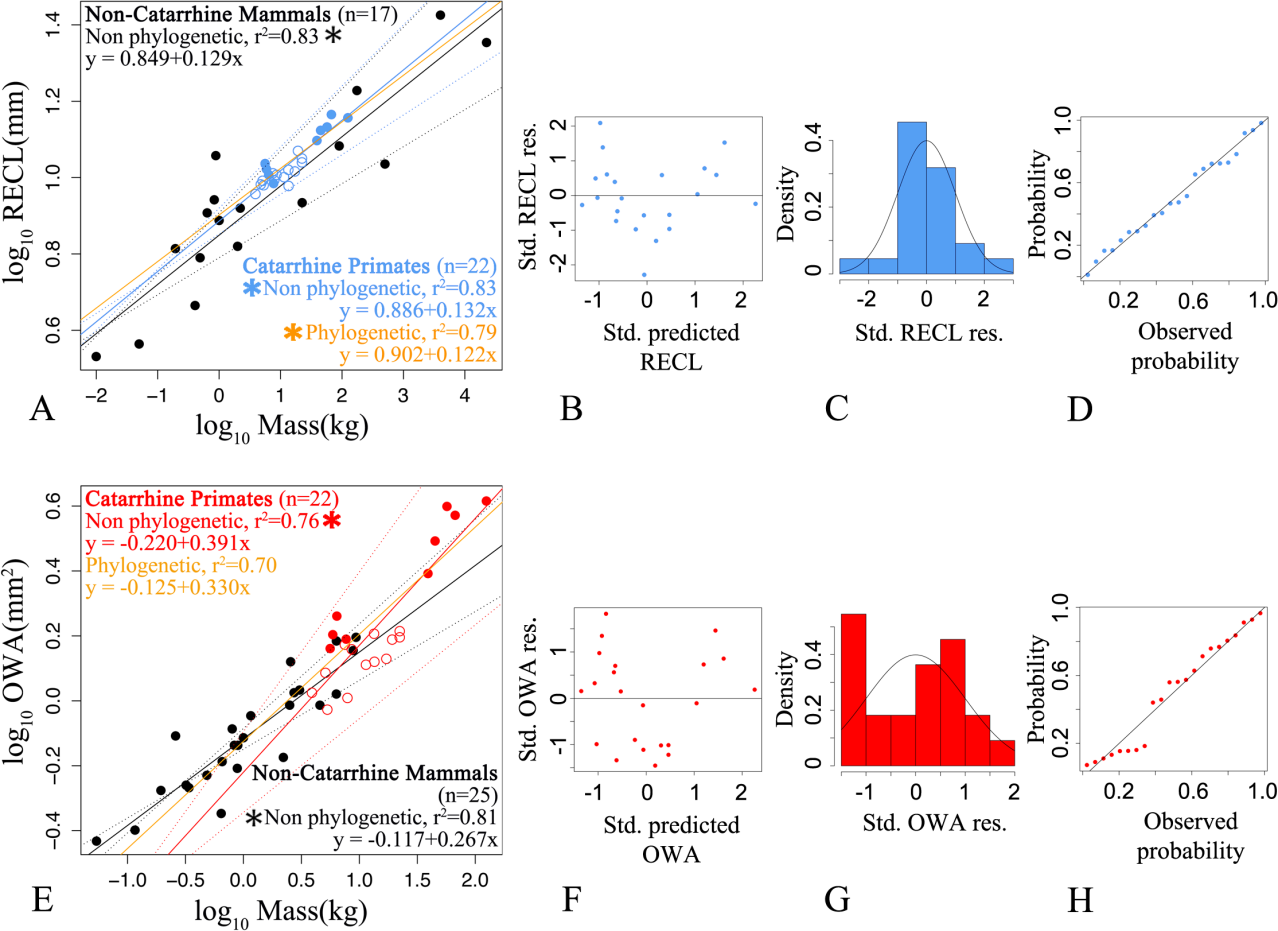
Bivariate non phylogenetic and phylogenetically controlled interspecies linear regressions between RECL, OWA and body mass. (A) RECL (in blue) versus body mass for hominoids (filled circles, n = 9 species mean values), cercopithecoids (open circles, n = 13 species mean values) and non-catarrhine mammal species mean values (black circles, 6 non-catarrhine primate species and 11 non-primate mammal species) (S4 and S5 Tables); dotted lines indicate 95% confidence regions for both the ordinary least squares (OLS) and the phylogenetically controlled regressions. (B) Plots of RECL standardized residuals (y-axis) against RECL standardized predicted values (x-axis) (S1 Text). (C). Histogram of RECL standardized residuals (S1 Text). (D) RECL probability-probability plots (S1 Text). (E) OWA (in red) versus body mass; symbols as in A (filled black circles represent mean values for 25 non-catarrhine primate species). (F,G,H) Residual plots as in (B,C,D). Both non phylogenetic and phylogenetically controlled regressions indicate that only increases of RECL (but not OWA) are the allometric correlates of body mass (which explains 79 to 83% and 70 to 81% of cochlear variation among catarrhine or non-catarrhine primate species). Significant correlation is indicated by * (P≤0.05).

We next investigate the role of body mass (with its well known high phylogenetic signal in mammals [10,51]) in driving correlated trait evolution among apes. We examine the relationship between cochlear features based on the residuals from associations between each trait and body size. In non-phylogenetic controlled regressions, the model with the best AIC is obtained with RECL alone. Non-phylogenetic and phylogenetic controlled regressions indicate that increases of only ECL and RECL are the allometric correlates of body mass in hominoid, cercopithecoid and non-catarrhine mammal species (Fig 5, S4 and S5 Tables). Body mass explains 75–87% and 83–90% of the observed ECL and RECL variation, respectively. Accounting for phylogeny does not affect the estimates of the slopes (S4 and S5 Tables).

Importantly, the absence of phylogenetic signal in the correlations between each cochlear trait and body mass (S5 Table) indicates that the residuals are also independent of phylogeny. In same-sized animals, the non-catarrhine RECL (with its smaller intercept) is shorter than the catarrhine one.

Residual analyses reveal that modern humans and two *Hylobates* species display a larger RECL given their mean body mass while two cercopithecoid species show the reverse trend (S1 Text, S6 Table). Phylogenetically controlled regressions indicate that OWA, TUR and CUR are independent on body mass. Adding TUR to the predictors of ECL or OWA to the predictors of RECL do not improve the model fit (S4 and S5 Tables).

Only the RECL and OWA mean values obtained in *Homo sapiens* (14.6 and 3.73, respectively), *Homo erectus* (14.2 and 3.3, respectively) and *Paranthropus robustus* (14.3 and 4.1, respectively) are much higher (with at least two standard deviations above the observed modern human mean; S1 Table) than those expected for their body mass with phylogenetically controlled linear regressions in catarrhine species (respectively 13.3 and 3.0, 12.6 and 2.6, 12.4 and 2.5) (Fig 5). The premodern (*Homo erectus*) and modern human cochleae set apart from at least contemporaneous non-human primates and australopiths because they show a RECL and OWA larger than expected for their body mass.

### Predictions of ancestral cochlear features

**Fig 6:**
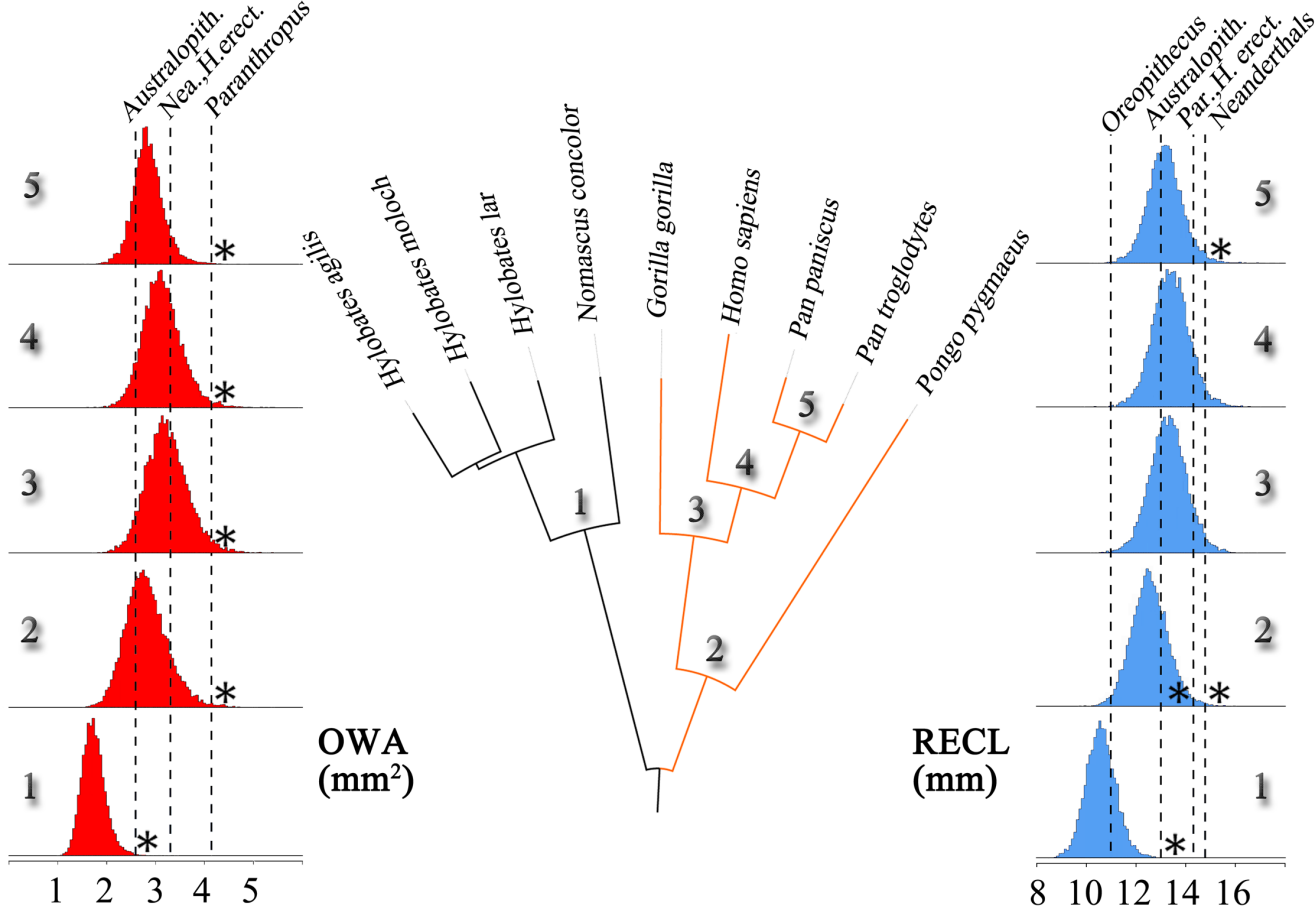
Posterior distributions of ancestral states (S1 Text, S7 Table) for OWA (in red) and RECL (in blue) at each internal node of the hominoid gene-based phylogenetic tree (show in the center). The hominoid most recent common ancestor is not represented. The ancestral reconstructions are compared with the actual mean values obtained through micro-ct measurements on the fossil *Oreopithecus bambolii*, *Australopithecus sp*., *Paranthropus robustus*, *Homo erectus* and Neanderthals. Significant deviations of each fossil value from each posterior distribution is calculated with Z-scores (S7 Table) and indicated by * (P≤0.05).

Results for RECL and OWA reconstructed ape ancestral states [18,19] at all internal nodes show narrower ranges when we consider the catarrhine tree rather than the hominoid one (S7 Table). This result confirms that uncertainties inherent to ancestral node reconstructions are expected to decrease with the number of taxa involved or the phylogenetic distance between the tip data and ancestral nodes [52]. We could not improve the ancestral state reconstructions by incorporating fossil data because the *Oreopithecus* phylogenetic status is much debated [36] and all the other fossil cochleae are on the hominin branch. Both RECL and OWA ancestral reconstructions show increased values on the hominid clade from its MRCA onwards, except in the *Pan* MRCA with its slightly decreased values (Fig 6). The *Hylobates*/*Nomascus* MRCA values are much smaller than in the hominid clade and in the hominoid MRCA (Fig 6).

We compare the RECL and OWA ancestral states with fossil values (Fig 6; S1 and S7 Tables). The *Oreopithecus* short RECL (OWA not preserved) does not fit any MRCA on the great ape clade but instead fit the hylobatid MRCA (Fig 6). All the fossil hominin RECLs fall within the African hominid MRCAs distributions (only the Neanderthal RECL value fall outside the *Pan* MRCA distribution) and cannot be differentiated from the moden human variation sampled in this study (Fig 3). With the exception of *Paranthropus*, all the fossil hominin OWAs fall within the four hominid MRCA distributions. In line with our underestimation of the *Paranthropus* OWA (from a phylogenetically controlled linear regression discussed above) which is not discernable from either *Pongo*, *Gorilla* and modern human values (Fig 4), we observe that it nevertheless fall well above any hominid MRCAs distributions (Fig 6).

## Discussion

### Which gross cochlear features can be used to investigate auditory capacities among catarrhine species?

As yet, there is no consensus as to which gross cochlear variable best predicts auditory capacities (e.g., refs 32,37). Given the complexity of the mechanics of the mammalian cochlea, it would be simplistic to argue that a handful of morphological cochlear features allow us to make precise predictions about hearing. This is beyond the scope of this study. Our goal is to investigate the phylogenetic signal of cochlear features that have been considered as reliable indicators of auditory capacities but, at the same time, that can be taxonomically useful among apes’ main lineages. In this context, since we find that RECL is taxonomically more useful as compared to ECL and TUR alone, it is also important to clarify the physiological significance of RECL. West (1985) [32] demonstrates that basilar membrane length (here approximated by ECL) is strongly related to the upper and lower limits of hearing in ground dwelling mammals, while the number of spiral turns (here approximated by TUR) is more associated with the octave range of audible frequencies. However, West (1985) [32] also emphasizes that “taken together, spiral turns and basilar membrane length can be used to predict the absolute values of the upper and lower limits of hearing and octave range better than either alone.” (op.cit., p. 1100). Further comparative data [29] confirmed that a combination of ECL with TUR (here represented by RECL) “resulted in significantly better correlations of observed and expected hearing limits in generalized terrestrial mammals, in particular for the low frequency limit of hearing.” (op.cit., p. 8). Therefore, we argue in favor of the use of RECL as a more useful taxonomic discriminator and auditory predictor than either ECL or TUR considered alone.

Since the cochlear spiral shape (here approximated by CUR) has been considered to show the strongest correlation with low frequency hearing [37], here we also use this parameter with the same measurement method as in Manoussaki et al. (2008) [37]. However, our results are not in line with those reported in Manoussaki et al. (2008). Contrary to this previous study, our measurements on a much larger sample of extant humans and catarrhine species show that human CUR values do not depart from other mammal CUR data (Fig 4). We therefore doubt that CUR is taxonomically useful even though it may discriminate species with unique low-frequency limits.

### The cochlear phylogenetic signal among ape species

Whenever possible, one should compute the phylogenetic signal by using mean species values with an assessment of intraspecific variability (which is never negligible). In the present study, we obtain data sets that consist of several measurements for some species but only one per species for others. This represents an important difficulty to be taken into account in order to assess errors due to uncertain species means. Phylogenetic comparative methods that incorporate intraspecific variability are relatively new and have not been used in this study [53,54]. Our calculated cochlear phylogenetic signals among apes’ species may be underestimated as already demonstrated when variability in the data is attributed solely to the between-species component (as opposed to the within-species component) [55]. However, in this case, the ancestral state estimations are unaffected [55]. Moreover, our cochlear phylogenetic signals among apes’ species are likely not significantly biased, as is the case when one uses log-transformed data and when the intraspecific variances are distributed homogeneously among species with respect to the tree [53–55].

The RECL and OWA parameters were the most useful to explain the variability measured in our sample of catarrhine cochleae, but we obtained a significantly high phylogenetic signal for RECL only. Therefore we expect higher levels of homoplasy for OWA than for RECL among catarrhine species. In the absence of similar measures of the phylogenetic signal in other primate groups, we cannot generalize our results beyond catarrhines. Insight to cochlear evolution may be gained by comparing the amount of phylogenetic signal of its features between catarrhines and other primate subclades. Previous studies investigated phylogenetically controlled interspecific allometries of ECL and OWA among non-hominoid primates (including some cercopithecoid species) [56,57]. However, to the best of our knowledge, our study is the first to provide measures of the strength of the phylogenetic signal of RECL and OWA in primate species. Since the distinction between the phylogenetic noise of homoplasy from the phylogenetic signal of homology is central for accurate phylogenetic reconstructions, the significantly high phylogenetic signal measured for RECL (but not for OWA) lead us to consider only this cochlear feature as particularly useful for the identification of monophyletic groups and for the reconstruction of ancestral states among catarrhines. However, since RECL evolution may have deviated from Brownian motion (i.e., close relatives may be more similar than expected under Brownian motion evolution), our measure of its phylogenetic signal will need to be confirmed by further tests using more species [10].

It was demonstrated that behavioral traits showed significantly lower phylogenetic signal than did any other trait type such as morphology or body mass [10]. Indeed, behavioral traits such as vocal communication may be more evolutionary malleable (i.e., highly adaptive) because distantly related species occupying similar habitats may possess hearing patterns more similar than those of closely related populations in different habitats [58]. For instance, species occupying dense vegetation habitats tend to have lower frequencies sensitivities because longer wavelengths are less attenuated by the scattering effects of leaves and branches. Therefore, ecological and behavioral parameters can cause sensitivity to frequencies to be similar by convergent evolution or chance, thus limiting their usefulness for inferring phylogeny.

### Correlated trait evolution and interspecies allometry

Changes in adult body sizes may have also driven apes’ cochlear evolution. It has long been observed that the highest audible frequency for most mammal species is negatively correlated with body mass (to the exception of humans among primates). It was also suggested that OWA evolutionary changes among non-hominoid primates were largely associated with overall increases in body size [57]. Coleman and Boyer (2012) observed that body mass explained 82.5% of their observed OWA variation [57]. Our non-phylogenetic linear regressions presented here using samples of catarrhines (n = 22) and non-catarrhines (n = 25) are partly in line with those of Coleman and Boyer (2012) obtained mainly using a different sample of primate species. When we consider our phylogenetically adjusted results, evolutionary changes in body mass appear closely related to RECL but not to OWA (Fig 5). Therefore, differences in OWA interspecific allometries may exist between primate sub-clades. More data are needed to clarify this issue. Coleman and Boyer (2012) observed that their sample of living haplorrhine species had relatively longer cochlea than in other primates [57]. In our study, we also observed that in same-sized animals, the non-catarrhine RECL was shorter than the catarrhine one. Moreover, adding TUR to the predictors of ECL or adding OWA to the predictors of RECL did not improve the allometric models fit (S4 and S5 Tables). These findings did not support the hypotheses that the cochlear spiral form represented a mean of economically housing a lengthened duct into a smaller space with putative tradeoffs between cochlear elongation and optimal coiling [37].

Our phylogenetically adjusted investigations of the interspecies cochlear allometries revealed the absence of phylogenetic signal and indicated that the residuals were independent of phylogeny, hence more likely evolutionary malleable (i.e., highly adaptive). Residual analyses revealed that modern humans and two *Hylobates* species displayed a larger RECL given their mean body mass while two cercopithecoid species showed the reverse trend (S3 Table). This suggested that the modern human and hylobatid cochlea may be better suited to process either lower frequencies, or a higher range of sounds.

The *Oreopithecus* skeleton (with its still debated phylogenetic relationships) preserves a large number of primitive features. For instance, it shows an extremely short face more closely resembling hylobatids than hominids, but a more hominid-like postcranial anatomy. On the basis of its overal cranial anatomy, *Oreopithecus* is mostly considered as representing a distinct, primitive hominoid clade which could be related to the European Dryopithecids [17]. Its inner ear morphology has been interpreted as very close to the living great ape and the *Dryopithecus brancoi* late Miocene conditions (see review in [36]). However, some of the *Oreopithecus* features are « most parsimoniously interpreted as either homoplasies or retained primitive hominid features » [20]. We found that the *Oreopithecus* short RECL fitted better the hylobatid MRCA than any hominid MRCA. Since we also found that this cochlear feature had a significantly high phylogenetic signal and was unlikely homoplasious, our results are well in line with previous interpretations that *Oreopithecus* represents a stem hominid with RECL values close to the hominoid MRCA due to smaller body mass than in great apes.

The premodern (*Homo erectus* and Neanderthals) and modern human cochleae setted apart from at least living non-human primates and australopiths because they showed a RECL and OWA larger than expected for their body mass. In this regard, our underestimated values for *Paranthropus* might be due to an underestimation of body mass in *Paranthropus* because its body mass estimates are still uncertain. In the absence of evidence for a significantly higher body mass in *Paranthrop*us (though it is not universally accepted [59]), functional adaptations for hearing sensitivity may have caused the considerable increase of OWA within this genus (4.1 mm^2^) [11], a value much higher than expected for its currently estimated mean body mass [37] (2.5 mm^2^).

Pending more evidence on, first, the neurohistology of the apes’ auditory cortex, second the interspecies allometric relationships between RECL, the length of the basilar membrane and the cochlear innervation density, we can as yet only discuss our results on cochlear allometry in the broader context of an increase in brain size during hominin evolution leading to a relatively large human brain as expected for its body mass [60]. Since the the human brain is a linearly scaled-up ape brain in its relationship between brain size and number of neurons [61] our observed disproportionate (larger than expected for body mass) premodern and modern human RECL and OWA may be associated by the increase of the neural bases for cochlear innervation occurring 2 Myrs ago. Indeed, the innervation density of the cochlear afferent and efferent fibers from base to apex appears very similar in living humans and chimpanzees [62]. Therefore, the human disproportionate cochlear features may be simply scaled with the number of neurones reaching the modiolar axis of the cochlea. Due to the sparce earliest human fossil record, inferences must be made cautiously. With the premodern *Homo* sample, we observe for the first time that hominin cranial capacity expands beyond the range of variation seen among great apes, with an increase of approximately 30% from *Australopithecus* [63–65]. However, at the same time, an average increase in body mass of approximately 30% also occurred [64,65].

### Intraspecific cochlear variation among apes

We found that combined-sex OWA CVs were much higher in the most sexually dimorphic hominoids (*Gorilla* and *Pongo*) only. If we assume that the underlying cause for increased combined-sex CVs is the result of higher between-sex size differences rather than within-sex variance, our results may indicate that sexual selection on body size has directly or indirectly caused larger OWAs in *Gorilla* and *Pongo* males. However, since males of these two dimorphic genera (with non dominant males being less size-dimorphic than dominant males) tend to be more variable than females [50], analyses examining unambiguous and molecularly sexed specimens are needed to test further whether the skeletal proxies of hearing capacities are sexually more dimorphic in the largest *Gorilla* and *Pongo* polygynous apes’ genera, independent or not of body mass. This might be the case since the production of otoacoustic emissions (OAEs) by the cochlea differs between the sexes in rhesus and marmoset monkeys, and in humans [66].

### Toward a more integrated study of auditory differences among apes

Apes comparative data on cochlear gross morphology, basic function and neural control in relation with auditory capacities are very limited. It is therefore impossible to interpret phylogenetic signals of cochlear features in a more integrated anatomical and functional framework. It has been demonstrated that ape skeletal features were more frequently homoplastic than soft tissues in primate and human evolution [67]. Tonotopic fields in the primate auditory cortex are interpreted as homologies [68], with a highly consistent relationship between the functional tonotopic maps of the primary auditory cortex and the underlying unique anatomical shape of Heschl's gyrus in humans [69,70]. Because the sulcal pattern is related to the onset of neuronal connectivity [71], it is well possible that the computational properties of the auditory cortex differ between non-human primates and humans. In addition to comparisons of the neural bases of hearing among apes, one of the most promising studies to develop in order to assess the underlying causes of differences in hearing capabilities among apes and humans is the cochlear amplifier, which can be studied non-invasively using otoacoustic emissions. The study of the evolutionary ecology of apes’ auditory sensitivity would greatly benefit from the use of otoacoustic emissions as a complement of behaviorally derived audiograms that are currently available only for common chimpanzees among the great apes (see review in [12]).

## Conclusions

Our proposed conceptual framework developed a model-based approach to simultaneously estimate the evolutionary history and phylogenetic signal of the cochlea among apes. We conclude that the evolutionary history of cochlear elongation in apes, a proxy of hearing capabilities, occurred mainly, but not only through body mass-dependent and non-homoplasious changes, an evidence which could be useful to improve classifications of fossil hominid species into true monophyletic groups.

Our predictions of ape’s hearing evolution made from measurements of phylogenetic signals, phylogenetically controlled interspecies allometries, ancestral states reconstructions and comparisons with the fossil record indicate that, in few cases, cochlear elongation occurred through tradeoffs between body mass increase and some factors yet to be identified. We were unable to identify them in this study. However, our approach newly established the high phylogenetic signal of cochlear morphological features among apes and portrayed the uniqueness of the “hypertrophied” premodern and modern human cochleae as compared to their ancestral states. As compared to early hominins, the relatively longer human basilar membrane would be consistent with an increased low-frequency sensitivity [29,32] and suggest that cochlear changes in early member of the genus *Homo* may have facilitated an increased emphasis on longer-range communication signals. More detailed and integrated studies will help to clarify the respective roles of phylogeny and natural selection on phenotypes favoring shifts in hearing ranges and sound localization abilities during apes’ evolution.

## Supporting Information

S1 Fig Principal component analysis of all five cochlear features investigated in this study and measured among hominoid living species and fossil taxa. (DOCX)

S1 Text The Akaike information criterion, residual analyses and ancestral states reconstructions. (DOCX)

S1 Table Living and fossil specimens with individual and combined-sex mean species values for cochlear micro-ct measurements and body mass estimates for living species only. (DOCX)

S2 Table Likelihood ratio tests to determine, for each cochlear trait and body mass taken separately in catarrhines, cercopithecoid and hominoid species, which model best fitted our data: a standard Brownian constant-variance random-walk model or a directional random-walk model. (PDF)

S3 Table Phylogenetic signal (as represented by Pagels’ λ) calculated to obtain a value that maximizes the likelihood of the data for each cochlear trait and body mass taken separately in catarrhines, cercopithecoid and hominoid species, under a Brownian model of evolution. We report likelihood tests for significant departure of λ from 0 and 1.
(PDF)

S4 Table Results for the non-phylogenetic bivariate and multivariate linear regressions to investigate the relationship between log-transformed mean species values for cochlear parameters and body mass (BM). (PDF)

S5 Table Results for the phylogenetically adjusted bivariate and multivariate linear regressions to investigate the relationship between log-transformed mean species values for cochlear parameters and body mass (BM). Likelihood ratio (LR) tests made between the two best models.
(PDF)

S6 Table Standardized residual values for non-phylogenetic bivariate linear correlations (OLS) between RECL or OWA, and body mass. (PDF)

S7 Table Reconstructions of the distributions of RECL and OWA ancestral states at all the internal nodes in the hominoid and catarrhine phylogenies compared with fossil values using Z-scores and p-values (left or right-tailed) using the catarrhine tree only. (PDF)
