# Supplementary material for: Disproportionate Cochlear Length in Genus Homo Shows a High Phylogenetic Signal during Apes’ Hearing Evolution
Source: PLoS One. 2015 Jun 17;10(6):e0127780. doi: 10.1371/journal.pone.0127780 (PMC4471221; doi:10.1371/journal.pone.0127780)
Supplement: S1 Fig — (DOCX) [file pone.0127780.s001.docx]

**Supporting Information**

**Figure S1**

Principal component analysis of all five cochlear features investigated in this study and measured among hominoid living species and fossil taxa.
