# Supplementary material for: Disproportionate Cochlear Length in Genus Homo Shows a High Phylogenetic Signal during Apes’ Hearing Evolution
Source: PLoS One. 2015 Jun 17;10(6):e0127780. doi: 10.1371/journal.pone.0127780 (PMC4471221; doi:10.1371/journal.pone.0127780)
Supplement: S1 Text — (DOCX) [file pone.0127780.s002.docx]

**Supporting Information**

**Method S1**

The Akaike information criterion (AIC)

We used two distinct approaches to get meaningful inter-species allometric information out of our data : the p-values of linear regressions and the Akaike information criterion (AIC). The AIC method does not provide a test of a model in the sense of testing a [null hypothesis](http://en.wikipedia.org/wiki/Null_hypothesis). Given a set of candidate models for the data, the preferred model is the one with the minimum AIC value. Hence, it not only rewards goodness of fit, but it includes a penalty that is an increasing function of the number of estimated parameters. This penalty discourages [overfitting](http://en.wikipedia.org/wiki/Overfitting) (increasing the number of free parameters in the model improves the goodness of the fit, regardless of the number of free parameters in the data-generating process). To apply AIC in practice, we start with a set of candidate models. We then find the models' corresponding AIC values. As show in the table below, the model with the best AIC was obtained with RECL alone.

| Candidate models | AIC |
| --- | --- |
| ECL + TUR + RECL + TAP + OWA | -58.37 |
| TUR + RECL + TAP + OWA | -61.4 |
| RECL + TAP + OWA | -65.07 |
| RECL + TAP | -68.26 |
| RECL | -71.05 |

Residual analyses

Residual analyses were used to discriminate between species that had cochlear values deviating from the catarrhine inter-species allometric plan. We evaluated graphically how well the non-phylogenetic linear bivariate models fitted the data and how the data met the assumptions of the linear model. To evaluate deviations from the linear model assumptions we examined various diagnostic plots.

We first graphed the standardized residuals (y-axis) (Fig.4 B and F) against standardized predicted values (x-axis) and added optional horizontal line to aid in interpretation.  The linearity assumption was here supported for both RECL and OWA because the amount of points scattered above and below the line was equal. For RECL, the points above the line were underpredicted (*Hylobates lar*, *Homo sapiens* and *Hylobates* agilis showed a large RECL given their mean body mass) while the the ones below the line were overpredicted (*Macaca sylvanus*, *Papio cynocephalus* show a small RECL given their mean body weight). For OWA, the points above the line were underpredicted (*Hylobates moloch*, *Pongo pygmaeus* and *Hylobates* agilis show a large OWA given their mean body weight) while the ones below the line were overpredicted (*Papio cynocephalus, Piliocolobus badius, Papio ursinus and Macaca sylvanus* show a small OWA given their mean body mass). The homogeneity of variance assumption was not supported for RECL and OWA because the vertical scatter was not the same across all standardized predicted values.

We then graphed a histogram to assess the assumption that the residuals were normally distributed (Fig.4 C and G). The RECL residuals matched better the normal distribution than the OWA residuals. Finally, we graphed a probability-probability plot to assess the assumption that the residuals were normally distributed (Fig.4 D and H). The abline (0,1) function was used to draw a diagonal line across the plot for comparison purposes. Again, the RECL distribution was considered much closer to normality because the RECL plotted points matched better the diagonal line than the OWA ones.

Ancestral states reconstructions

The posterior probability of the phylogenetic tree given the data matrix (X), is noted “Pr [Tree, τ, υ, θ | Data, X]” and is given by:

$$Pr[Tree, \tau, \upsilon, \theta| Data, X]= \frac{Pr[Data, X \left| Tree, \tau, \upsilon, \theta\right] x\Pr\left[ Tree, \tau, \upsilon, \theta\right]}{Pr[Data, X]}$$

The distribution Pr [Tree, τ, υ, θ] specifies the prior probability of the tree parameter values before the observations have been made (the 50% majority consensus gene-based tree provided by the 10kTrees Website). Pr [Data, X | Tree, τ, υ, θ] is the likelihood function and describes the probability of the data matrix (X) under different tree parameter values.

The posterior probability involves a summation over all trees and, for each tree, integration over all possible combinations of its parameters (τ, υ, θ). This integral is difficult to evaluate because the tree parameters vary continuously. Alternatively, Pr [Tree, τ, υ, θ | Data, X] can be evaluated by Markow chain Monte Carlo (MCMC) methods (Gilks et al. 1996). A Markow chain was constructed in which new values of the parameters were proposed on successive iterations of the Markow chain. At each step in the chain, models were accepted or rejected by the Metropolis-Hasting algorithm (Metropolis et al. 1953, Hastings 1970). The chain was allowed to run to convergence for a large number of iterations. All MCMC chains ran for 2 000 000 iterations with sampling every 100 iterations and with a burn-in of 200 000. The rate deviation setting was adjusted so that acceptance values ranged between 0.2 and 0.4. The changes to the ancestral state were accepted between 20-40% of the time.

Results for the ECL, RECL, OWA and BW ancestral states at all internal nodes showed narrower ranges when we considered the catarrhine tree rather than the hominoid one (Table S10). This result confirmed that uncertainties inherent to ancestral node reconstructions were expected to increase with the number of taxa involved or the phylogenetic distance between the tip data and ancestral nodes (Martins 1999).

**Supplementary References**

Begall S, Burda H (2006) Acoustic communication and burrow acoustics are reflected in the ear morphology of the coruro (*Spalacopus cyanus*, Octodontidae), a social fossorial rodent. J Morphol 267: 382–390.

Braga J, Thackeray JF, Dumoncel J, Descouens D, Bruxelles L *et al* (2013) A new partial temporal bone of a juvenile hominin from the site of Kromdraai B (South Africa). J Hum Evol 65: 447-456.

Burda H, Ulehlova L, Branis M (1984) Morphology of the middle and inner ear in *Panthera* species - *P. tigris* and *P. onca* (Felidae, Carnivora, Mammalia). Vest Cs Spolec Zool 48: 9–14.

Coleman M, Ross C (2004) Primate auditory diversity and its influence on hearing performance. Anat. Rec*.* 281A: 1123-1137.

Echteler SM, Fay RR, Popper AN (1994) Structure of the mammalian cochlea. In: Fay RR, Popper AN, editors. Comparative Hearing: Mammals*.* New York: Springer. pp 134-171.

Gilks WR, Richardson S, Spiegelhalter D (1996) Markov Chain Monte Carlo in Practice. London: Chapman & Hall.

Metropolis N, Rosenbluth AW, Rosenbluth MN, Teller AH, Teller E (1953). [Equations of state calculations by fast computing machines](http://en.wikipedia.org/wiki/Equations_of_State_Calculations_by_Fast_Computing_Machines). [J Chem Phys](http://en.wikipedia.org/wiki/Journal_of_Chemical_Physics) 21: 1087-1092.

Hastings WK (1970) Monte Carlo sampling methods using Markov chains and their applications. Biometrika 57: 97-109.

Ketten D (1992) in The evolutionary biology of hearing (eds Webster D, Fay R, Popper A) 717-750 (Springer).

Ketten D (2000) in *Hearing by whales and dolphins.* (Ed. Fay, R.) 43-108 (Springer).

Kirk E, Gosselin-Ildari A (2009) Cochlear labyrinth volume and hearing abilities in primates. Anat. Rec*.* 292: 765-776.

Martins EP (1999) Estimation of ancestral states of continuous characters: a computer simulation study. Syst Biol 48: 642-650.

Martinez I *et al* (2004) Auditory capacities in Middle Pleistocene humans from the Sierra de Atapuerca in Spain. Proc. Natl. Acad. Sci. USA 101: 9976-9981.

Parks S, Ketten D, O’Malley J, Arruda J (2007) Anatomical predictions of hearing in the North Atlantic right whale. Anat Rec 290: 734-744.

Rook L, Bondioli L, Casali F, Rossi M, Köhler M *et al* (2004) The bony labyrinth of *Oreopithecus bambolii*. J Hum Evol 46: 349-356.

Smith R, Jungers W (1997) Body mass in comparative primatology. J Hum Evol 32: 523–559.

West CD (1985) The relationship of the spiral turns of the cochlea and the length of the basilar membrane to the range of audible frequencies in ground dwelling mammals. J Acoust Soc Am 77: 1091–1101.
