# Supplementary material for: Disproportionate Cochlear Length in Genus Homo Shows a High Phylogenetic Signal during Apes’ Hearing Evolution
Source: PLoS One. 2015 Jun 17;10(6):e0127780. doi: 10.1371/journal.pone.0127780 (PMC4471221; doi:10.1371/journal.pone.0127780)
Supplement: S1 Table — (DOCX) [file pone.0127780.s003.docx]

**Supporting Information**

**Table S1**

Living and fossil specimens with individual and combined-sex mean species values for cochlear micro-ct measurements and body mass (BM) estimates for living species only (since the modern human sample was of Northern European origin, estimates were taken for Danish as the geographically closest sample). Mass data in fossil species are discussed in the text. Specimens are curated in the following institutions: DNMNH (Ditsong National Museum of Natural History, Pretoria, South Africa; formerly Transvaal Museum), IANPS (Institut d’Anatomie Normale et Pathologique de Strasbourg, France), MHNT (Muséum d’Histoire Naturelle de Toulouse, France), MRCA (Musée Royal de l’Afrique Centrale, Tervuren, Belgium), MZS (Musée Zoologique, Strasbourg, France), SENCK (Senckenberg Forschungsinstitut und Naturmuseum, Frankfurt, Germany), UT-PS (Université de Toulouse, Paul Sabatier, France), WITS (Evolutionary Studies Institute, University of the Witwatersrand, Johannesburg, South Africa). F, Female; M, Male; L, Left; R, Right; U, Unknown; SD, Standard Deviation; CV, Coefficient of Variation; Min, Minimum; Max, Maximum. Sex attributions are not considered for fossil specimens. Voxel sizes are indicated in microns. Unless specified, all ECL, TUR and CUR measurements were taken on the left side. When left and right sides were available, we measured both oval window areas and we then calculated the mean value (in mm²). Previous published sources are: Braga *et al*. [1]; Rook *et al*. (2004) [2]; Martinez *et al.* (2004) [3]; Smith and Jungers (1997) [4]; Coleman and Ross (2004) [5]; West (1985) [6]; Echteler *et al.* ([1994](http://onlinelibrary.wiley.com/doi/10.1002/ar.20907/full#bib12)) [7]; Kirk and Gosselin-Ildari (2009) [8]; Ketten ([1992](http://onlinelibrary.wiley.com/doi/10.1002/ar.20907/full#bib25)) [9]; Parks *et al.* (2007) [10]; Ketten (2000) [11]; Burda *et al.* (1984) [12]; Begall and Burda (2006) [13]. * *Papio cynocephalus kindae*

| Specimens / Species with their  body mass mean values (BM) | Sex | Institutions | Micro-ct systems | Voxel size  (isometric) | ECL | TUR | RECL | CUR | OWA | | |
| --- | --- | --- | --- | --- | --- | --- | --- | --- | --- | --- | --- |
|  |  |  |  |  |  |  |  |  | Left | Right | Mean |
| Hominoid living species (n = 9) and fossil taxa (n = 5) | | | | | | | | | | | |
| *Homo sapiens sapiens* (n=22) (BM: 67.1) [4] | | | | |  |  |  |  |  |  |  |
| Embr 249 | U | IANPS | XtremeCT | 41.0 | 38.8 | 2.8 | 13.9 | 2.8 | 3.18 | 3.25 | 3.22 |
| Embr 323 | U | IANPS | XtremeCT | 41.0 | 39.8 | 2.9 | 13.7 | 3 | 3.93 | 3.98 | 3.96 |
| Embr 168 | M | IANPS | XtremeCT | 41.0 | 40.1 | 2.8 | 14.3 | 3 | 3.87 | 3.87 | 3.87 |
| Embr 308 | F | IANPS | XtremeCT | 41.0 | 41.7 | 2.7 | 15.4 | 2.7 |  | 3.63 |  |
| Embr 583 | F | IANPS | XtremeCT | 41.0 | 40.8 | 2.7 | 15.1 | 2.7 | 3.57 | 3.48 | 3.53 |
| Embr 385 | F | IANPS | XtremeCT | 41.0 | 38.5 | 2.6 | 14.8 | 2.4 | 3.30 | 3.49 | 3.40 |
| Embr 513 | F | IANPS | XtremeCT | 41.0 | 40.8 | 2.7 | 15.1 | 2.5 |  | 4.25 |  |
| Embr 576 | F | IANPS | XtremeCT | 41.0 | 40.8 | 2.8 | 14.6 | 2.6 | 4.25 |  |  |
| Embr 382 | F | IANPS | XtremeCT | 41.0 | 38 | 2.8 | 13.6 | 2.9 |  | 3.70 |  |
| Embr 281 | F | IANPS | XtremeCT | 41.0 | 42.4 | 2.9 | 14.6 | 2.7 | 3.69 | 3.99 | 3.84 |
| Embr 384 | F | IANPS | XtremeCT | 41.0 | 43 | 2.8 | 15.4 | 2.7 |  | 3.39 |  |
| Embr 121 | F | IANPS | XtremeCT | 41.0 | 36.3 | 2.6 | 14.0 | 2.6 |  | 3.39 |  |
| Embr 212 | F | IANPS | XtremeCT | 41.0 | 40.6 | 2.9 | 14.0 | 2.9 |  | 3.33 |  |
| Embr 277 | M | IANPS | XtremeCT | 41.0 | 41.2 | 2.8 | 14.7 | 2.7 | 3.66 | 3.84 | 3.75 |
| Embr 215 | M | IANPS | XtremeCT | 41.0 | 37.9 | 2.6 | 14.6 | 2.1 |  | 3.60 |  |
| Embr 388 | M | IANPS | XtremeCT | 41.0 | 40.7 | 2.7 | 15.1 | 2.5 | 3.61 | 3.82 | 3.72 |
| Embr 383 | M | IANPS | XtremeCT | 41.0 | 38.9 | 2.8 | 13.9 | 2.9 |  | 3.42 |  |
| Embr 479 | M | IANPS | XtremeCT | 41.0 | 40.8 | 2.7 | 15.1 | 2.6 | 3.89 | 3.87 | 3.88 |
| Embr 205 | M | IANPS | XtremeCT | 41.0 | 41.1 | 2.8 | 14.7 | 2.9 |  | 3.59 |  |
| Embr 473 | M | IANPS | XtremeCT | 41.0 | 44.7 | 2.9 | 15.4 | 3.2 | 4.62 | 4.64 | 4.63 |
| Embr 136 | M | IANPS | XtremeCT | 41.0 | 37.2 | 2.5 | 14.9 | 2.4 |  | 3.71 |  |
| Embr 179 | M | IANPS | XtremeCT | 41.0 | 37.9 | 2.5 | 15.2 | 2.3 |  | 4.04 |  |
| Mean (SD) | | | | | 40.1 (2.0) | 2.7 (0.1) | 14.6 (0.6) | 2.7 (0.3) |  |  | 3.73 (0.35) |
| CV | | | | | 0.05 | 0.04 | 0.04 | 0.11 |  |  | 0.09 |
| Min, Max | | | | | 36.3, 44.7 | 2.5, 2.9 | 13.6, 15.4 | 2.1, 3.2 |  |  | 3.22, 4.63 |
| Mean (SD) in Males (n=10) (BM: 72.1) [4]* | | | | | 40.1 (2.2) | 2.7 (0.1) | 14.7 (0.5) | 2.7 (0.3) |  |  | 3.82 (0.33) |
| CV in Males | | | | | 0.05 | 0.04 | 0.04 | 0.11 |  |  | 0.09 |
| Min, Max in Males | | | | | 37.2, 44.7 | 2.5, 2.9 | 13.6, 15.4 | 2.1, 3.2 |  |  | 3.42, 4.63 |
| Mean (SD) in Females (n=10) (BM: 62.1) [4]* | | | | | 40.3 (2.1) | 2.8 (0.1) | 14.7 (0.6) | 2.7 (0.2) |  |  | 3.67 (0.35) |
| CV in Females | | | | | 0.05 | 0.04 | 0.04 | 0.07 |  |  | 0.09 |
| Min, Max in Females | | | | | 36.3/43.0 | 2.6/2.9 | 13.6/15.4 | 2.4/2.9 |  |  | 3.33/4.25 |
|  | | | | |  |  |  |  |  |  |  |
| *Neanderthal* (n=2) | | | | |  |  |  |  |  |  |  |
| Kr-38.20 |  | www.nespos.org | | 40.0 | 38.8 | 2.5 | 15.5 | 2.3 |  |  |  |
| Kr-39.23 |  | www.nespos.org | | 40.0 | 40.6 | 2.9 | 14.0 | 3.2 |  |  |  |
| Mean (SD) | | | | | 39.7 | 2.7 | 14.7 | 2.7 |  |  | 3.4 [3] |
|  |  |  |  |  |  |  |  |  |  |  |  |
| *Homo erectus* |  |  |  |  |  |  |  |  |  |  |  |
| SK 847 |  | DNMNH | [1] | 21.7 | 34.0 | 2.4 | 14.2 | 2.2 |  |  | 3.32 |
|  |  |  |  |  |  |  |  |  |  |  |  |
| *Paranthropus robustus* (n=3) |  |  |  |  |  |  |  |  |  |  |  |
| TM 1517* |  | DNMNH | BIR ACTIS 225/300 | 45.6 | 36.6 | 2.6 | 14.4 | 2.4 |  |  | - |
| Sk 879 |  | DNMNH | [1] | 9.2 | 39.1 | 2.6 | 14.8 | 2.6 |  |  | 4.3 |
| SkW 18 |  | DNMNH | [1] | 11.14 | 33.9 | 2.5 | 13.8 | 2.7 |  |  | 3.9 |
| Mean (SD) | | | | | 36.5 (2.6) | 2.6 (0.1) | 14.3 (0.5) | 2.6 (0.2) |  |  | 4.1 (0.3) (n=2) |
| Min, Max | | | | | 33.9, 39.1 | 2.5, 2.6 | 13.8, 14.8 | 2.4, 2.7 |  |  | 3.9, 4.3 |
|  |  |  |  |  |  |  |  |  |  |  |  |
| *Australopithecus sp.* (n=4) |  |  |  |  |  |  |  |  |  |  |  |
| STS 5 |  | DNMNH | Metris X-Tek XT H225L | 76.15 | 38.7 | 2.7 | 14.3 | 2.9 |  |  | - |
| StW 329 |  | WITS | [1] | 33.1 | 32.1 | 2.7 | 11.8 | 2.0 |  |  | 2.0 |
| StW 98 |  | WITS | [1] | 33.1 | 33.4 | 2.6 | 12.7 | 3.1 |  |  | 3.1 |
| StW 255 |  | WITS | [1] | 33.1 | 35.7 | 2.8 | 12.8 | 2.8 |  |  | 2.8 |
| Mean (SD) | | | | | 35.0 (2.9) | 2.7 (0.1) | 12.9 (1.0) | 2.7 (0.5) |  |  | 2.4 (0.5) (n=3) |
| Min, Max | | | | | 32.1, 38.7 | 2.6, 2.8 | 11.8, 14.3 | 2.0,3.1 |  |  | 1.8, 2.7 |
|  |  |  | |  |  |  |  |  |  |  |  |
| *Oreopithecus bambolii* (n=1) |  |  | |  |  |  |  |  |  |  |  |
| BAC 208 |  | [2] | | 29.1 | 30.7 | 2.8 | 11.0 | 2.6 |  |  | - |
|  |  |  |  |  |  |  |  |  |  |  |  |
| *Pan paniscus* (n=7) (BM: 39.1) [4] |  |  |  |  |  |  |  |  |  |  |  |
| 84036 M6 |  | MRAC | XtremeCT | 41.0 | 33.8 | 2.8 | 12.1 | 2.9 |  | 2.16 |  |
| 29051 |  | MRAC | XtremeCT | 41.0 | 35.1 | 2.8 | 12.5 | 2.9 |  | 2.63 |  |
| 29054 |  | MRAC | Optiv CT160 | 41.0 | 38 | 2.9 | 13.1 | 3.3 |  | 2.88 |  |
| 84036 M8 |  | MRAC | Optiv CT160 | 8.0 | 40.8 | 2.9 | 14.1 | 3.2 |  | 2.54 |  |
| 26990 |  | MRAC | XtremeCT | 41.0 | 34.5 | 2.8 | 12.3 | 3 |  | 2.31 |  |
| 29007 |  | MRAC | XtremeCT | 41.0 | 32.2 | 2.8 | 11.5 | 2.8 |  | 2.37 |  |
| 29003 |  | MRAC | XtremeCT | 41.0 | 33.5 | 2.8 | 12.0 | 2.9 |  | 2.39 |  |
| Mean (SD) | | | | | 35.4 (3.0) | 2.8 (0.0) | 12.5 (0.8) | 3.0 (0.2) |  |  | 2.47 (0.24) |
| CV | | | | | 0.08 | 0.0 | 0.07 | 0.07 |  |  | 0.10 |
| Min, Max | | | | | 32.2, 40.8 | 2.8, 2.9 | 11.5, 14.1 | 2.8, 3.3 |  |  | 2.16, 2.88 |
|  |  |  |  |  |  |  |  |  |  |  |  |
| *Pan troglodytes* (n=9) (BM: 45.0) [4] |  |  |  |  |  |  |  |  |  |  |  |
| 044 |  | UT-PS | XtremeCT | 41.0 | 36.8 | 3 | 12.3 | 3.3 |  | 3.00 |  |
| 045 KG11 |  | UT-PS | XtremeCT | 41.0 | 36 | 2.8 | 12.9 | 2.8 |  | 3.85 |  |
| ZOO 2011.0.3 |  | MHNT | XtremeCT | 41.0 | 39.2 | 3.1 | 12.6 | 3.4 | 2.73 | 2.88 | 2.81 |
| 10732 |  | MRAC | XtremeCT | 41.0 | 38.6 | 3 | 12.9 | 3.1 |  | 3.12 |  |
| 072 |  | UT-PS | XtremeCT | 41.0 | 38.6 | 2.9 | 13.3 | 3.1 | 3.52 | 3.59 | 3.56 |
| 070 |  | UT-PS | XtremeCT | 41.0 | 41.9 | 2.9 | 14.4 | 2.8 |  | 2.94 |  |
| 071 |  | UT-PS | XtremeCT | 41.0 | 39.1 | 2.9 | 13.5 | 3 | 3.14 | 2.99 | 3.07 |
| RG 2538 |  | MRAC | XtremeCT | 41.0 | 38.9 | 2.9 | 13.4 | 3.4 | 2.66 | 2.74 | 2.70 |
| RG 7054 |  | MRAC | XtremeCT | 41.0 | 40.4 | 2.8 | 14.4 | 3.1 | 3.16 | 2.73 | 2.95 |
| Mean (SD) | | | | | 38.8 (1.7) | 2.9 (0.1) | 13.3 (0.8) | 3.1 (0.2) |  |  | 3.11 (0.37) |
| CV | | | | | 0.04 | 0.03 | 0.06 | 0.06 |  |  | 0.12 |
| Min, Max | | | | | 36.0, 41.9 | 2.8, 3.1 | 12.3,14.4 | 2.8, 3.4 |  |  | 2.70, 3.85 |
|  |  |  |  |  |  |  |  |  |  |  |  |
| *Gorilla gorilla* (n=7) (BM: 124.7) [4] |  |  |  |  |  |  |  |  |  |  |  |
| ZOO 2011.0.2 |  | MHNT | XtremeCT | 41.0 | 42.2 | 2.9 | 14.6 | 3 | 5.43 | 5.28 | 5.36 |
| ZOO 2011.0.5 |  | MHNT | XtremeCT | 41.0 | 45 | 3 | 15.0 | 3.6 | 3.87 | 3.55 | 3.71 |
| 068 |  | UT-PS | XtremeCT | 41.0 | 38.7 | 2.7 | 14.3 | 2.9 | 3.03 | 3.27 | 3.15 |
| 73018 M2 |  | MRAC | XtremeCT | 41.0 | 37.1 | 2.7 | 13.7 | 2.5 |  | 3.91 |  |
| ZOO 2011.0.4 |  | MHNT | XtremeCT | 41.0 | 37.9 | 2.8 | 13.5 | 2.6 | 4.31 | 4.07 | 4.19 |
| 9406 |  | MRAC | Optiv CT160 |  | 41.8 | 2.9 | 14.4 | 2.8 |  | 4.25 |  |
| 12282 |  | MRAC | XtremeCT | 41.0 | 38.7 | 2.6 | 14.9 | 2.7 |  | 4.34 |  |
| Mean (SD) | | | | | 40.2 (2.9) | 2.8 (0.1) | 14.4 (0.5) | 2.9 (0.4) |  |  | 4.13 (0.68) |
| CV | | | | | 0.07 | 0.04 | 0.04 | 0.14 |  |  | 0.16 |
| Min, Max | | | | | 37.1, 45.0 | 2.6, 3.0 | 13.5, 15.0 | 2.5, 3.6 |  |  | 3.15, 5.36 |
|  |  |  |  |  |  |  |  |  |  |  |  |
| *Pongo pygmaeus* (n=8) (BM: 57.0) [4] |  |  |  |  |  |  |  |  |  |  |  |
| ZOO 2011.0.8 |  | MHNT | XtremeCT | 41.0 | 35.1 | 2.9 | 12.1 | 3.2 | 3.29 | 3.40 | 3.35 |
| 5593 |  | MRAC | XtremeCT | 41.0 | 35.9 | 2.4 | 15.0 | 2.7 |  | 3.56 |  |
| 2625 |  | MZS | XtremeCT | 41.0 | 33.7 | 2.5 | 13.5 | 2.6 |  | 4.18 |  |
| 2627 |  | MZS | XtremeCT | 41.0 | 32.4 | 2.3 | 14.1 | 2.3 | 5.43 | 5.26 | 5.35 |
| 2629 |  | MZS | XtremeCT | 41.0 | 37.2 | 2.7 | 13.8 | 3 | 5.46 | 5.22 | 5.34 |
| 1576 |  | SENCK | Optiv CT160 | 12.2 | 32 | 2.5 | 12.8 | 2.8 |  | 2.80 |  |
| 6782 |  | SENCK | Optiv CT160 | 12.0 | 38.3 | 2.8 | 13.7 | 2.8 |  | 3.26 |  |
| 2630 |  | MZS | XtremeCT | 41.0 | 38.4 | 2.8 | 13.7 | 2.9 |  | 3.96 |  |
| Mean (SD) | | | | | 35.4 (2.5) | 2.6 (0.2) | 13.6 (0.8) | 2.8 (0.3) |  |  | 3.97 (0.94) |
| CV | | | | | 0.07 | 0.08 | 0.06 | 0.11 |  |  | 0.24 |
| Min, Max | | | | | 32.0, 38.4 | 2.3, 2.9 | 12.1, 15.0 | 2.3, 3.2 |  |  | 2.80, 5.35 |
|  |  |  |  |  |  |  |  |  |  |  |  |
| *Nomascus concolor* (n=1) (BM: 7.7) [4] |  |  |  |  |  |  |  |  |  |  |  |
| ZOO 2011.0.7 |  | MHNT | XtremeCT | 41.0 | 28 | 2.9 | 9.7 | 3 | 1.83 | 1.27 | 1.55 |
|  |  |  |  |  |  |  |  |  |  |  |  |
| *Hylobates moloch* (n=1) (BM: 6.4) [4] |  |  |  |  |  |  |  |  |  |  |  |
| 2633 |  | MZS | XtremeCT | 41.0 | 28.5 | 2.8 | 10.2 | 2.9 | 1.80 | 1.85 | 1.83 |
|  |  |  |  |  |  |  |  |  |  |  |  |
| *Hylobates lar* (n=1) (BM: 5.6) [4] |  |  |  |  |  |  |  |  |  |  |  |
| 59164 |  | SENCK | Optiv CT160 | 9.0 | 29.4 | 2.7 | 10.9 | 2.8 |  | 1.45 |  |
|  |  |  |  |  |  |  |  |  |  |  |  |
| *Hylobates agilis* (n=2) (BM: 5.9) [4] |  |  |  |  |  |  |  |  |  |  |  |
| 3196 |  | SENCK | Optiv CT160 | 7.3 | 30.3 | 2.9 | 10.4 | 2.8 |  | 1.60 |  |
| 2635 |  | MZS | Optiv CT160 | 8.3 | 32.9 | 3.1 | 10.6 | 3.4 |  | 1.60 |  |
| Mean (SD) | | | | | 31.6 (1.8) | 3.0 (0.1) | 10.5 (0.1) | 3.1 (0.4) |  | 1.60 (0) |  |
|  |  |  |  |  |  |  |  |  |  |  |  |
| *Hylobates sp.* (n=4) |  |  |  |  |  |  |  |  |  |  |  |
| Mean (SD) | | | | | 30.3 (1.9) | 2.9 (0.2) | 10.5 (0.3) | 3.0 (0.3) |  |  | 1.62 (0.16) |
| CV | | | | | 0.06 | 0.06 | 0.03 | 0.10 |  |  | 0.10 |
| Min, Max | | | | | 28.5, 32.9 | 2.7, 3.1 | 10.2, 10.9 | 2.8, 3.4 |  |  | 1.45, 1.83 |
| Cercopithecoid living species (n = 13) | | | | | | | | | | | |
| *Papio hamadryas* (n=2) (BM: 13.4) [4] |  |  |  |  |  |  |  |  |  |  |  |
| 682 |  | MHNT | XtremeCT | 41.0 | 36.4 | 3.4 | 10.7 | 3.6 |  | 1.53 |  |
| 494 |  | MHNT | XtremeCT | 41.0 | 30.8 | 3 | 10.3 | 3.5 |  | 1.69 |  |
| Mean (SD) | | | | | 33.6 (4.0) | 3.2 (0.3) | 10.5 (0.3) | 3.6 (0.1) |  | 1.61 (0.11) |  |
|  |  |  |  |  |  |  |  |  |  |  |  |
| *Papio cynocephalus* (n=5) (BM: 17.1) [4] |  |  |  |  |  |  |  |  |  |  |  |
| 052 LUNHO |  | UT-PS | XtremeCT | 41.0 | 32.7 | 3.3 | 9.9 | 3.5 |  | 1.34 |  |
| 31230 |  | MRAC | XtremeCT | 41.0 | 29 | 3.1 | 9.4 | 3.3 |  | 1.15 |  |
| 3502 * |  | MRAC | Optiv CT160 | 8.00 | 33 | 3 | 11.0 | 2.4 |  | 1.36 |  |
| 3498 * |  | MRAC | XtremeCT | 41.0 | 30.3 | 2.8 | 10.8 | 3 |  | 1.54 |  |
| 4151 * |  | MRAC | Optiv CT160 | 7.8 | 30.3 | 2.8 | 10.8 | 3 |  | 1.35 |  |
| Mean (SD) | | | | | 31.1 (1.7) | 3.0 (0.2) | 10.4 (0.7) | 3.0 (0.4) |  |  | 1.35 (0.14) |
| CV | | | | | 0.06 | 0.07 | 0.07 | 0.14 |  |  | 0.10 |
| Min, Max | | | | | 29.0 / 33.0 | 2.8 / 3.3 | 9.4 / 11.0 | 2.4 / 3.5 |  |  | 1.15 / 1.54 |
|  |  |  |  |  |  |  |  |  |  |  |  |
| *Papio ursinus* (n=1) (BM: 22.3) [4] |  |  |  |  |  |  |  |  |  |  |  |
| 73034 M4 |  | MRAC | XtremeCT | 41.0 | 32.9 | 3 | 11.0 | 3.6 |  | 1.57 |  |
|  |  |  |  |  |  |  |  |  |  |  |  |
| *Papio anubis* (n=2) (BM: 19.2) [4] |  |  |  |  |  |  |  |  |  |  |  |
| 8637 |  | MRAC | Optiv CT160 | 8.6 | 34.1 | 2.9 | 11.8 | 3 |  | 1.63 |  |
| 11553 |  | MRAC | Optiv CT160 | 8.1 | 37.6 | 3.2 | 11.8 | 3.4 |  | 1.46 |  |
| Mean (SD) | | | | | 35.9 (2.5) | 3.1 (0.2) | 11.8 (0) | 3.2 (0.3) |  | 1.55 (0.12) |  |
|  |  |  |  |  |  |  |  |  |  |  |  |
| *Mandrillus sphinx* (n=1) (BM: 22.3) [4] |  |  |  |  |  |  |  |  |  |  |  |
| 503 |  | MHNT | XtremeCT | 41.0 | 34.8 | 3.1 | 11.2 | 3 |  | 1.64 |  |
|  |  |  |  |  |  |  |  |  |  |  |  |
| *Macaca radiate* (n=1) (BM: 5.3) [4] |  |  |  |  |  |  |  |  |  |  |  |
| OST.AC 506 |  | MHNT | XtremeCT | 41.0 | 27.7 | 2.9 | 9.6 | 3.3 | 0.89 | 0.98 | 0.94 |
|  |  |  |  |  |  |  |  |  |  |  |  |
| *Macaca sylvanus* (n=2) (BM: 13.5) [4] |  |  |  |  |  |  |  |  |  |  |  |
| OST.AC.490 |  | MHNT | XtremeCT | 41.0 | 29.7 | 3.1 | 9.6 | 3.2 | 1.31 | 1.29 | 1.30 |
| OST.AC 427 |  | MHNT | XtremeCT | 41.0 | 25.5 | 2.7 | 9.4 | 3 | 1.34 | 1.31 | 1.33 |
| Mean (SD) | | | | | 27.6 (3.0) | 2.9 (0.3) | 9.5 (0.1) | 3.1 (0.1) |  |  | 1.32 (0.02) |
|  |  |  |  |  |  |  |  |  |  |  |  |
| *Cercopithecus mona* (n=1) (BM: 5.1) [4] |  |  |  |  |  |  |  |  |  |  |  |
| 28608 |  | MRAC | XtremeCT | 41.0 | 31.4 | 3.2 | 9.8 | 3.2 |  | 1.22 |  |
|  |  |  |  |  |  |  |  |  |  |  |  |
| *Cercopithecus hamlyni* (n=1) (BM: 3.9) [4] |  |  |  |  |  |  |  |  |  |  |  |
| 28411 |  | MRAC | XtremeCT | 41.0 | 29 | 3.2 | 9.1 | 3.2 |  | 1.06 |  |
|  |  |  |  |  |  |  |  |  |  |  |  |
| *Cercocebus torquatus* (n=1) (BM: 7.5) [4] |  |  |  |  |  |  |  |  |  |  |  |
| 73018 M389 |  | MRAC | XtremeCT | 41.0 | 30.8 | 3 | 10.3 | 2.9 |  | 1.49 |  |
|  |  |  |  |  |  |  |  |  |  |  |  |
| *Colobus angolensis* (n=3) (BM: 8.6) [4] |  |  |  |  |  |  |  |  |  |  |  |
| 10746 |  | MRAC | Optiv CT160 | 7.0 | 30.5 | 3.2 | 9.5 | 3 |  | 1.18 |  |
| 11556 |  | MRAC | XtremeCT | 41.0 | 29.5 | 2.8 | 10.5 | 2.8 | 1.47 | 1.59 | 1.53 |
| 15447 |  | MRAC | XtremeCT | 41.0 | 30.3 | 2.9 | 10.4 | 2.9 | 1.58 | 1.65 | 1.62 |
| Mean (SD) | | | | | 30.1 (0.5) | 3.0 (0.2) | 10.2 (0.6) | 2.9 (0.1) |  |  | 1.44 (0.23) |
| CV | | | | | 0.02 | 0.07 | 0.05 | 0.03 |  |  | 0.16 |
| Min, Max | | | | | 29.5, 30.5 | 2.8, 3.2 | 9.5, 10.5 | 2.8, 3.0 |  |  | 1.18, 1.62 |
|  |  |  |  |  |  |  |  |  |  |  |  |
| *Colobus guereza* (n=4) (BM: 11.4) [4] |  |  |  |  |  |  |  |  |  |  |  |
| 8425 |  | MRAC | XtremeCT | 41.0 | 30.2 | 2.9 | 10.4 | 3.1 |  | 1.58 |  |
| 8441 |  | MRAC | XtremeCT | 41.0 | 29.3 | 2.8 | 10.5 | 3 | 1.25 |  |  |
| 10646 |  | MRAC | XtremeCT | 41.0 | 28.1 | 2.9 | 9.7 | 2.7 | 1.15 |  |  |
| 10747 |  | MRAC | XtremeCT | 41.0 | 27.7 | 2.9 | 9.6 | 2.7 | 1.16 | 1.22 | 1.19 |
| Mean (SD) | | | | | 28.8 (1.1) | 2.9 (0.1) | 10.0 (0.5) | 2.9 (0.2) |  |  | 1.29 (0.20) |
| CV | | | | | 0.04 | 0.02 | 0.05 | 0.07 |  |  | 0.15 |
| Min, Max | | | | | 27.7, 30.2 | 2.8, 2.9 | 9.6, 10.5 | 2.7, 3.1 |  |  | 1.15, 1.58 |
|  |  |  |  |  |  |  |  |  |  |  |  |
| *Piliocolobus badius* (n=4) (BM: 7.9) [4] |  |  |  |  |  |  |  |  |  |  |  |
| 10099 |  | MRAC | XtremeCT | 41.0 | 28.1 | 2.7 | 10.4 | 2.5 | 1.09 | 0.97 | 1.03 |
| 28402 |  | MRAC | XtremeCT | 41.0 | 27.9 | 2.7 | 10.3 | 2.6 | 0.95 | 1.05 | 1.00 |
| 28403 |  | MRAC | XtremeCT | 41.0 | 26.9 | 2.8 | 9.6 | 2.5 | 1.05 | 1.05 | 1.05 |
| 10289 |  | MRAC | XtremeCT | 41.0 | 26.2 | 2.9 | 9.0 | 2.3 | 0.95 | 1.06 | 1.01 |
| Mean (SD) | | | | | 27.3 (0.9) | 2.8 (0.1) | 9.8 (0.7) | 2.5 (0.1) |  |  | 1.02 (0.02) |
| CV | | | | | 0.03 | 0.03 | 0.07 | 0.05 |  |  | 0.02 |
| Min, Max | | | | | 26.2, 28.1 | 2.7, 2.9 | 9.0, 10.4 | 2.3, 2.6 |  |  | 1.00, 1.05 |
| Non-catarrhine primate living species (n = 26) [5] | | | | | | | | | | | |
| Alouatta sp. (BM: 6.345) | | | | |  |  |  |  |  |  | 1.53 |
| *Aotus sp.* (BM: 1) | | | | | 22.4 | 2.9 | 7.7 |  |  |  | 0.77 |
| *Ateles* sp. (BM: 9.37) | | | | |  |  |  |  |  |  | 1.57 |
| *Brachyteles sp.* (BM: 8.84) | | | | |  |  |  |  |  |  | 1.43 |
| *Cacajao* sp. (BM: 3.05) | | | | |  |  |  |  |  |  | 1.08 |
| *Callicebus* sp. (BM: 1.158) | | | | |  |  |  |  |  |  | 0.90 |
| *Callimico sp.* (BM: 0.484) | | | | |  |  |  |  |  |  | 0.59 |
| *Callithrix jacchus* (BM: 0.32) | | | | |  |  |  |  |  |  | 0.55 |
| *Cebuella sp.* (BM: 0.116) | | | | |  |  |  |  |  |  | 0.40 |
| *Cebus sp.* (BM: 2.735) | | | | |  |  |  |  |  |  | 1.06 |
| *Chiropotes sp.* (BM: 2.507) | | | | |  |  |  |  |  |  | 0.97 |
| *Leontopithecus sp.* (BM: 0.655) | | | | |  |  |  |  |  |  | 0.65 |
| *Saguinus sp.* (BM: 0.643) | | | | |  |  |  |  |  |  | 0.45 |
| *Saimiri sp.* (BM: 0.885) | | | | | 25.7 | 2.25 | 11.4 |  |  |  | 0.62 |
| *Galago senegalensis* (BM: 0.194) | | | | | 17.6 | 2.7 | 6.5 |  |  |  | 0.53 |
| *Arctocebus sp.* (BM: 0.26) | | | | |  |  |  |  |  |  | 0.78 |
| *Nycticebus coucang* (BM: 0.64) | | | | | 18.6 | 2.3 | 8.1 |  |  |  |  |
| *Perodicticus potto* (BM: 0.835) | | | | | 21.0 | 2.4 | 8.8 |  |  |  | 0.73 |
| *Avahi sp.* (BM: 0.8) | | | | |  |  |  |  |  |  | 0.82 |
| *Daubentonia sp.* (BM: 2.555) | | | | |  |  |  |  |  |  | 1.32 |
| *Indri sp.* (BM: 6.335) | | | | |  |  |  |  |  |  | 1.05 |
| *Lemur catta* (BM: 2.21) | | | | | 20.8 | 2.5 | 8.3 |  |  |  | 0.67 |
| *Lepilemur sp.* (BM: 0.894) | | | | |  |  |  |  |  |  | 0.73 |
| *Microcebus sp.* (BM: 0.054) | | | | |  |  |  |  |  |  | 0.37 |
| *Phaner sp.* (BM: 0.339) | | | | |  |  |  |  |  |  | 0.54 |
| *Propithecus sp.* (BM: 4.575) | | | | |  |  |  |  |  |  | 0.97 |
| Non-primate mammal living species (n = 11) | | | | | | | | | | | |
| *Oryctolagus cuniculus* (BM: 2) [6,7 in 8] | | | | | 15.2 | 2.3 | 6.6 |  |  |  |  |
| *Eubalaena glacialis* (BM: 22500) [9 in 8, 10] | | | | | 55.6 | 2.46 | 22.6 |  |  |  |  |
| *Tursiops truncatus* (BM: 175) [9 in 8, 11] | | | | | 38.9 | 2.3 | 16.9 |  |  |  |  |
| *Felis catus* (BM: 22.5) [6,7 in 8] | | | | | 25.8 | 3 | 8.6 |  |  |  |  |
| *Pantera onca* (BM: 90) [12 in 8] | | | | | 33.3 | 2.75 | 12.1 |  |  |  |  |
| *Bos taurus* (BM: 500) [6,7 in 8] | | | | | 38 | 3.5 | 10.9 |  |  |  |  |
| *Cavia porcellus* (BM: 0.406) [7 in 8] | | | | | 18.5 | 4 | 4.6 |  |  |  |  |
| *Chinchilla laniger* (BM: 0.49) [6, 13 in 8] | | | | | 18.5 | 3 | 6.2 |  |  |  |  |
| *Meriones unguiculatus* (BM: 0.05) [7 in 8] | | | | | 12.1 | 3.3 | 3.7 |  |  |  |  |
| *Mus musculus* (BM: 0.01) [6,7 in 8] | | | | | 6.8 | 2 | 3.4 |  |  |  |  |
| *Elephas maximus* (BM: 4000) [6,7 in 8] | | | | | 60 | 2.25 | 26.7 |  |  |  |  |
