# Supplementary material for: Disproportionate Cochlear Length in Genus Homo Shows a High Phylogenetic Signal during Apes’ Hearing Evolution
Source: PLoS One. 2015 Jun 17;10(6):e0127780. doi: 10.1371/journal.pone.0127780 (PMC4471221; doi:10.1371/journal.pone.0127780)
Supplement: S2 Table — (PDF) [file pone.0127780.s004.pdf]

Supporting Information

Table S2

Likelihood ratio tests to determine, for each cochlear trait and body mass taken separately in catarrhines, cercopithecoid and hominoid species, which model best fitted our data: a standard Brownian constant-variance random-walk model with one parameter (variance of evolution, Var) or a directional random-walk model with two parameters (variance of evolution – Var - and a parameter that reflects the degree of directional change). * p-values < 0.05 indicate that the Brownian model best fitted our data (at the 5% level).

	Brownian constant-variance model			Directional random-walk model				p-value
	Lh	Alpha	Var	Lh	Alpha	Beta	Var	
Catarrhines (n=22)								
ECL	38.348	1.503	0.030	38.463	1.637	-0.687	0.029	0.632
TUR	53.035	0.457	0.008	53.787	0.284	0.889	0.007	0.220
RECL	38.437	1.046	0.030	39.123	1.368	-1.650	0.028	0.241
CUR	37.503	0.467	0.032	37.553	0.376	0.467	0.032	0.732
OWA	18.972	0.271	0.173	19.358	0.859	-3.020	0.167	0.380
BM	-1.325	1.202	1.098	-0.298	3.579	-12.207	1.000	0.152
Cercopithecoids (n=13)								
ECL	23.470	1.471	0.035	23.515	1.646	-1.313	0.035	0.764
TUR	33.731	0.474	0.007	37.188	-0.135	4.590	0.004	0.009
RECL	23.784	0.999	0.034	25.015	1.846	-6.381	0.028	0.117
CUR	20.481	0.472	0.056	21.046	-0.286	5.714	0.051	0.288
OWA	16.166	0.092	0.108	16.171	-0.013	0.792	0.108	0.920
BM	0.692	0.940	1.170	2.529	6.917	-45.027	0.882	0.055
Hominoids (n=9)								
ECL	16.141	1.528	0.032	16.196	1.428	0.90	0.031	0.740
TUR	20.987	0.443	0.011	20.990	0.457	-0.123	0.011	0.938
RECL	16.244	1.084	0.031	16.284	0.999	0.765	0.031	0.777
CUR	19.939	0.463	0.014	20.089	0.570	-0.966	0.013	0.584
OWA	6.658	0.413	0.261	6.873	-0.147	5.059	0.249	0.512
BM	-0.474	1.409	1.275	-0.456	1.769	-3.258	1.269	0.850
