# Supplementary material for: Disproportionate Cochlear Length in Genus Homo Shows a High Phylogenetic Signal during Apes’ Hearing Evolution
Source: PLoS One. 2015 Jun 17;10(6):e0127780. doi: 10.1371/journal.pone.0127780 (PMC4471221; doi:10.1371/journal.pone.0127780)
Supplement: S3 Table — We report likelihood tests for significant departure of λ from 0 and 1. (PDF) [file pone.0127780.s005.pdf]

Supporting Information

Table S3

Phylogenetic signal (as represented by Pagels' λ) calculated to obtain a value that maximizes the likelihood of the data for each cochlear trait and body mass taken separately in catarrhines, cercopithecoid and hominoid species, under a Brownian model of evolution. We report likelihood tests for significant departure of λ from 0 and 1. * indicates a phylogenetic signal not significantly different from λ=1 but significantly different from λ=0 (at 5%).

	Brownian model with λ = 1			Brownian model with λ = 0			Brownian model with λ at maximum likelihood (ML)				ML vs λ = 0	ML vs λ = 1
	Lh	Alpha	Var	Lh	Alpha	Var	Lh	Alpha	Var	Pagels' λ	p-value	p-value
Catarrhines (n=22)												
ECL	38.348	1.503	0.030	33.152	1.507	0.015	39.643	1.504	0.019	0.908*	0.000	0.108
TUR	53.035	0.457	0.008	50.432	0.465	0.003	55.039	0.459	0.003	0.715	0.002	0.045
RECL	38.437	1.046	0.030	30.324	1.042	0.019	40.043	1.046	0.019	0.931*	0.000	0.073
CUR	37.503	0.467	0.032	42.531	0.479	0.006	43.863	0.472	0.007	0.477	0.103	0.000
OWA	18.972	0.271	0.173	5.576	0.245	0.184	59.739	0.276	0.007	1.030	0.000	0.000
BW	-1.325	1.202	1.098	-12.287	1.173	0.935	99.937	1.203	0.000	1.033	0.000	0.000
Cercopithecoids (n=13)												
ECL	23.470	1.471	0.035	24.122	1.488	0.014	25.163	1.475	0.016	0.705	0.147	0.066
TUR	-	-	-	-	-	-	-	-	-	-	-	-
RECL	23.784	0.999	0.034	27.065	1.009	0.009	27.560	1.002	0.010	0.578	0.320	0.006
CUR	20.481	0.472	0.056	23.082	0.490	0.016	24.094	0.478	0.016	0.516	0.155	0.007
OWA	16.166	0.092	0.108	14.944	0.123	0.056	16.168	0.093	0.104	0.990	0.118	0.950
BW	0.692	0.940	1.170	-0.149	1.021	0.569	0.899	0.944	0.858	0.889	0.148	0.520
Hominoids (n=9)												
ECL	16.141	1.528	0.032	12.537	1.529	0.037	16.256	1.528	0.027	0.928*	0.006	0.632
TUR	20.987	0.443	0.011	-	-	-	23.485	0.447	0.003	0.000	-	0.025
RECL	16.244	1.084	0.031	11.866	1.081	0.042	16.317	1.084	0.034	1.044*	0.003	0.702
CUR	19.939	0.463	0.014	-	-	-	22.702	0.466	0.004	0.000	-	0.019
OWA	6.658	0.413	0.261	2.797	0.387	0.318	6.658	0.413	0.258	0.995*	0.005	1.000
BW	-0.474	1.409	1.275	-6.674	1.350	2.608	0.847	1.417	1.373	1.100*	0.000	0.104
