# Supplementary material for: Disproportionate Cochlear Length in Genus Homo Shows a High Phylogenetic Signal during Apes’ Hearing Evolution
Source: PLoS One. 2015 Jun 17;10(6):e0127780. doi: 10.1371/journal.pone.0127780 (PMC4471221; doi:10.1371/journal.pone.0127780)
Supplement: S4 Table — (PDF) [file pone.0127780.s006.pdf]

Supporting Information

Table S7

Results for the non-phylogenetic bivariate and multivariate linear regressions to investigate the relationship between log-transformed mean species values for cochlear parameters and body mass (BM). * indicates significant correlations (at the 5% level).

Dep./Indep	r	r^2	2,1-tailed p	OLS		RMA	
				Intercept	Slope	Intercept	Slope
Catarrhines (n=22)							
ECL/BM*	0.86	0.73	< 0.001*	1.378	/ 0.110	1.353	0.131
ECL/TUR&BM*	0.88	0.77	< 0.001*	-3.892	/ 2.942	-4.437	3.304
TUR/BM*	-0.43	0.18	0.049, 0.024*	0.496	/ -0.025	0.535	-0.059
RECL/BM*	0.91	0.83	< 0.001*	0.886	/ 0.132	0.870	0.146
CUR/BM	-0.14	0.02	0.541, 0.270	0.494	/ -0.012	0.521	-0.036
OWA/BM*	0.87	0.76	< 0.001*	-0.220	/ 0.391	-0.289	0.450
RECL/OWA&BM*	0.94	0.89	< 0.001*	-5.783	/ 5.888	-6.143	6.235
ECL/TUR	-0.14	0.02	0.547, 0.273	1.641	/ -0.292	3.673	-4.650
ECL/CUR	0.04	0.00	0.864, 0.432	1.477	/ 0.057	-6.955	17.62
ECL/OWA*	0.84	0.70	< 0.001*	1.449	/ 0.239	1.437	0.291
RECL/OWA*	0.94	0.88	< 0.001*	0.968	/ 0.303	0.964	0.324
RECL/CUR	-0.24	0.06	0.283, 0.141	1.228	/ -0.395	2.512	-3.068
TUR/CUR*	0.66	0.44	< 0.001*	0.251	/ 0.449	0.111	0.740
TUR/OWA*	-0.55	0.30	0.008, 0.004*	0.483	/ -0.073	0.496	-0.128
CUR/OWA	-0.24	0.06	0.289, 0.144	0.491	/ -0.047	0.504	-0.105
Cercopithecoids (n=13)							
ECL/BM*	0.61	0.37	0.027, 0.013 *	-4.857	/ 3.951	-8.705	6.537
ECL/TUR&BM*	0.71	0.50	0.007, 0.004*	-2.829	/ 2.231	-4.280	3.206
TUR/BM	-0.06	0.00	0.842, 0.421	1.419	/ -0.832	11.81	-22.48
RECL/BM*	0.81	0.66	< 0.001*	-5.623	/ 6.583	-7.529	8.472
CUR/BM	0.18	0.03	0.558, 0.279	0.493	/ 1.075	-5.743	13.785
OWA/BM*	0.76	0.57	0.003, 0.001*	0.723	/ 2.419	0.626	3.209
RECL/OWA&BM*	0.85	0.73	< 0.001*	-2.658	/ 2.772	-3.229	3.337
ECL/TUR*	0.65	0.42	0.017, 0.008*	0.019	/ 0.310	-0.224	0.474
ECL/CUR	0.45	0.20	0.122, 0.061	-0.235	/ 0.488	-0.770	0.848
ECL/OWA*	0.80	0.64	0.001, < 0.001*	-2.291	/ 1.623	-2.893	2.027
RECL/OWA*	0.78	0.61	0.002, < 0.001*	-1.880	/ 1.984	-2.569	2.667
RECL/CUR	0.18	0.03	0.554, 0.277	0.243	/ 0.245	-0.677	1.157
TUR/CUR*	0.57	0.33	0.040, 0.020*	-0.132	/ 1.297	-0.454	1.968
TUR/OWA	0.37	0.14	0.207, 0.103	-0.640	/ 1.589	-1.971	4.361
CUR/OWA	0.30	0.09	0.311, 0.156	-0.158	/ 0.572	-1.298	2.897
Hominoids (n=9)							
ECL/BM*	0.93	0.87	< 0.001*	1.381	/ 0.110	1.370	0.118
ECL/TUR&BM*	0.94	0.89	< 0.001*	-4.657	/ 3.437	-4.956	3.632

TUR/BM	-0.38	0.14	0.316, 0.158	0.465	-0.013	0.479	-0.024
RECL/BM*	.95	0.90	< 0.001*	0.917	0.121	0.909	0.127
CUR/BM	-.26	0.07	0.5036, 0.2518	0.479	-0.010	0.501	-0.027
OWA/BM*	.97	0.94	< 0.001*	-0.068	0.338	-0.084	0.350
RECL/OWA&BM*	0.95	0.91	< 0.001*	-6.278	6.372	-6.639	6.706
ECL/TUR	-.21	0.05	0.5802, 0.2901	1.849	-0.715	4.883	-7.507
ECL/CUR	-.16	0.03	0.6763, 0.3381	1.760	-0.496	4.363	-6.090
ECL/OWA*	.90	0.81	< 0.001*	1.411	0.306	1.398	0.338
RECL/OWA*	.95	0.89	< 0.001*	0.947	0.346	0.940	0.364
RECL/CUR	-.38	0.15	0.3102, 0.1551	1.667	-1.260	2.855	-3.813
TUR/CUR*	.87	0.76	0.002, 0.001*	0.076	0.798	0.029	0.898
TUR/OWA	-.46	0.21	0.2162, 0.1081	0.465	-0.046	0.475	-0.073
CUR/OWA	-.34	0.12	0.3715, 0.1857	0.480	-0.038	0.496	-0.080
Non-catarrhine mammals (RECL, n=17; OWA, n=25)							
RECL/BM*	0.91	0.83	< 0.001*	0.849	0.129	0.840	0.142
OWA/BM*	0.91	0.81	< 0.001*	-0.117	0.267	-0.118	0.298
All mammals (RECL, n=40; OWA, n=47)							
RECL/BM*	0.91	0.83	< 0.001*	0.869	0.133	0.856	0.146
OWA/BM*	0.94	0.88	< 0.001*	-0.119	0.304	-0.131	0.325
