# Supplementary material for: Disproportionate Cochlear Length in Genus Homo Shows a High Phylogenetic Signal during Apes’ Hearing Evolution
Source: PLoS One. 2015 Jun 17;10(6):e0127780. doi: 10.1371/journal.pone.0127780 (PMC4471221; doi:10.1371/journal.pone.0127780)
Supplement: S5 Table — Likelihood ratio (LR) tests made between the two best models. (PDF) [file pone.0127780.s007.pdf]

Supporting Information

Table S5

Results for the phylogenetically adjusted bivariate and multivariate linear regressions to investigate the relationship between log-transformed mean species values for cochlear parameters and body mass (BM). Likelihood ratio (LR) tests made between the two best models (i.e., models with the highest likelihoods).* indicates significant correlations (at the 5% level).

Dep./Indep.	λML	LRtest	Likelihood			r²			2-tailed p	Intercept (λ at ML)	Slope(s) (λ at ML)
			λ0	λ1	λML	λ0	λ1	λML			
Catarrhines (n=22)											
ECL/BW*	0	< .001	48.52	42.72	48.52	0.75	0.33	0.75	< 0.001	1.378	0.110
ECL/TUR&BW*	0.30	0.294	51.18	47.27	51.73	0.81	0.56	0.76	< 0.001	1.000	0.792, 0.120
RECL/BW*	0.32	0.081	50.25	45.75	51.77	0.84	0.49	0.79	< 0.001	0.902	0.122
OWA/BW	0.73	0.019	21.91	25.99	28.73	0.77	0.47	0.70	0.094	-0.125	0.330
RECL/OWA&BW*	0	< .001	57.61	46.28	57.61	0.92	0.51	0.92	< 0.001	0.929	0.193, 0.057
ECL/OWA*	0.57	0.053	46.70	44.29	48.57	0.71	0.42	0.62	< 0.001	1.435	0.266
RECL/OWA*	0	< .001	53.76	43.18	53.76	0.88	0.35	0.88	< 0.001	0.968	0.305
TUR/CUR*	0.67	0.046	57.06	57.70	59.70	0.45	0.35	0.35	< 0.001	0.294	0.351
Cercopithecoids (n=13)											
ECL/BW*	0.42	0.610	27.17	25.15	27.30	0.37	0.23	0.31	< 0.001	1.396	0.084
ECL/TUR&BW*	0	< .001	36.09	29.01	36.09	0.84	0.58	0.84	< 0.001	0.695	1.437, 0.101
RECL/BW*	0	< .001	34.07	27.12	34.07	0.66	0.40	0.66	< 0.001	0.907	0.100
OWA/BW	0	0.019	20.49	17.76	20.49	0.57	0.22	0.57	0.095	-0.119	0.237
RECL/OWA&BW*	0	< .001	35.32	27.17	35.32	0.72	0.42	0.72	< 0.001	0.925	0.153, 0.064
ECL/OWA*	0	0.004	30.79	26.65	30.79	0.64	0.39	0.64	< 0.001	1.439	0.395
RECL/OWA*	0	< .001	33.19	24.94	33.19	0.61	0.16	0.61	< 0.001	0.971	0.307
TUR/CUR*	0.70	0.290	36.33	35.71	36.27	0.33	0.26	0.21	< 0.001	0.381	0.200
Hominoids (n=9)											
ECL/BW*	0	0.015	21.59	18.65	21.59	0.87	0.44	0.87	< 0.001	1.380	0.110
ECL/TUR&BW*	0	0.030	22.19	19.85	22.19	0.89	0.59	0.89	0.004	1.137	0.524, 0.117
RECL/BW*	0	0.032	22.04	19.75	22.04	0.90	0.55	0.90	< 0.001	0.918	0.121
OWA/BW	0	0.022	15.04	12.42	15.04	0.94	0.73	0.94	0.202	-0.069	0.338
RECL/OWA&BW*	0	0.023	22.44	19.87	22.44	0.91	0.58	0.91	< 0.001	0.929	0.161, 0.067
ECL/OWA*	0	0.133	20.09	18.96	20.09	0.82	0.48	0.82	< 0.001	1.411	0.307
RECL/OWA*	0	0.030	21.83	19.48	21.83	0.89	0.53	0.89	< 0.001	0.947	0.345
TUR/CUR	0	0.549	29.84	29.66	29.84	0.76	0.86	0.76	0.382	0.074	0.801
