# Supplementary material for: Disproportionate Cochlear Length in Genus Homo Shows a High Phylogenetic Signal during Apes’ Hearing Evolution
Source: PLoS One. 2015 Jun 17;10(6):e0127780. doi: 10.1371/journal.pone.0127780 (PMC4471221; doi:10.1371/journal.pone.0127780)
Supplement: S6 Table — (PDF) [file pone.0127780.s008.pdf]

Supporting Information

Table S6

Standardized residual values for non-phylogenetic bivariate linear correlations (OLS) between RECL, OWA, and body mass. * indicates the most significant residual values.

Species	RECL	OWA
Homo_sapiens	1.525*	0.858
Pan_paniscus	0.037	-0.106
Pan_troglodytes	0.783	0.732
Gorilla_gorilla	-0.238	0.187
Pongo_pygmaeus	0.593	1.463*
Nomascus_concolor	-0.734	0.701
Hylobates_moloch	0.608	1.825*
Hylobates_lar	2.088*	0.977
Hylobates_agilis	1.386*	1.349*
Papio_hamadryas	-0.571	-0.150
Papio_cynocephalus	-1.306*	-1.452*
Papio_ursinus	-0.962	-1.219*
Papio_anubis	0.588	-1.017
Mandrillus_sphinx	-0.556	-1.011
Macaca_radiata	-0.064	-0.989
Macaca_sylvanus	-2.283*	-1.111*
Cercopithecus_mona	0.493	0.327
Cercopithecus_hamlyni	-0.274	0.156
Cercocebus_torquatus	0.394	0.562
Colobus_angolensis	-0.082	0.150
Colobus_guereza	-0.974	-0.897
Piliocolobus_badius	-0.454	-1.337
