# Supplementary material for: Disproportionate Cochlear Length in Genus Homo Shows a High Phylogenetic Signal during Apes’ Hearing Evolution
Source: PLoS One. 2015 Jun 17;10(6):e0127780. doi: 10.1371/journal.pone.0127780 (PMC4471221; doi:10.1371/journal.pone.0127780)
Supplement: S7 Table — (PDF) [file pone.0127780.s009.pdf]

Supporting Information

Table S7

Reconstructions of the distributions of RECL and OWA ancestral states at all the internal nodes in the hominoid and catarrhine phylogenies compared with fossil values using Z-scores and p-values (left or right-tailed) using the catarrhine tree only. * indicates significant deviations from the ancestral state distribution (at the 5% level).

	RECL														
Nodes	Catarrhine tree					Hominoid tree					Z-scores (p-values) for comparisons using the catarrhine tree				
	Quantiles 0.5%	Quantiles 2.5%	Mean	Quantiles 97.5%	Quantiles 99.5%	Quantiles 0.5%	Quantiles 2.5%	Mean	Quantiles 97.5%	Quantiles 99.5%	*Oreopithecus*	*Australopithecus*	*Paranthropus*	*Homo erectus*	Neanderthals
Homo/Pan	11.44	11.96	13.40	14.90	15.54	10.86	11.72	13.47	15.31	16.23	-3.33 (< 0.01)	-0.69 (0.25)	-1.2 (0.12)	1.11 (0.133)	1.81 (0.035)
Pan	11.29	11.78	13.13	14.62	15.23	10.64	11.43	13.13	15.04	16.13	-3.16 (< 0.01)	-1.34 (0.37)	-1.57 (0.06)	1.59 (0.056)	2.33 (< 0.01)
(Homo(Pan))Gorilla	11.28	11.84	13.30	14.80	15.42	10.91	11.72	13.43	15.23	16.19	-3.15 (< 0.01)	-0.55 (0.29)	-1.33 (0.09)	1.23 (0.109)	1.92 (0.027)
Homo/Great Apes	10.73	11.19	12.56	14.06	14.67	10.69	11.27	12.79	14.50	15.29	-2.28 (0.011)	-0.45 (0.33)	-2.32 (0.01*)	2.39 (0.008)	3.12 (< 0.01)
Hylobatids	9.02	9.44	10.59	11.86	12.41	8.78	9.29	10.68	12.28	13.04	0.71 (0.239)	-3.63 (< 0.01*)	-5.84 (< 0.01*)	6.28 (< 0.01)	7.15 (< 0.01)
Hominoids	10.00	10.44	11.75	13.15	13.69						-1.15 (0.125)	-1.64 (< 0.05*)	-3.64 (< 0.01*)	3.74 (< 0.01)	4.50 (< 0.01)
Cercopithecoids	8.69	9.12	10.37	11.69	12.20						1.01 (0.156)	-3.83 (< 0.01*)	-5.95 (< 0.01*)	6.13 (< 0.01)	6.93 (< 0.01)
	OWA														
Nodes	Catarrhine tree					Hominoid tree					Z-scores (p-values) for comparisons using the catarrhine tree				
	Quantiles 0.5%	Quantiles 2.5%	Mean	Quantiles 97.5%	Quantiles 99.5%	Quantiles 0.5%	Quantiles 2.5%	Mean	Quantiles 97.5%	Quantiles 99.5%		*Australopithecus*	*Paranthropus*	*Homo erectus*	
Homo/Pan	2.26	2.48	3.20	4.06	4.45	1.73	2.15	3.23	4.62	5.51		-1.39 (0.08)	3.06 (< 0.02*)	0.28 (0.390)	
Pan	2.12	2.32	2.92	3.61	4.02	1.60	2.00	3.00	4.42	5.36		-0.73 (0.23)	4.60 (< 0.01*)	1.27 (0.102)	
(Homo(Pan))Gorilla	2.21	2.48	3.27	4.20	4.62	1.85	2.20	3.30	4.78	5.68		-1.44 (0.07)	-1.78 (0.04*)	0.08 (0.468)	
Homo/Great Apes	1.88	2.10	2.85	3.81	4.32	1.80	2.07	3.00	4.22	4.90		-0.4 (0.34)	-2.60 (< 0.01*)	1.20 (0.115)	
Hylobatids	1.21	1.32	1.73	2.26	2.50	1.02	1.23	1.85	2.70	3.22		-2.52 (< 0.01*)	-8.94 (< 0.01*)	7.66 (< 0.01)	
Hominoids	1.52	1.68	2.32	3.13	3.53							-0.94 (0.17)	-4.39 (< 0.01*)	3.06 (< 0.01)	
Cercopithecoids	0.91	1.01	1.44	1.98	2.21							-4.67 (< 0.01*)	-9.85 (< 0.01*)	8.65 (< 0.01)	
